# Influence of IL28B Polymorphisms on Response to a Lower-Than-Standard Dose peg-IFN-α 2a for Genotype 3 Chronic Hepatitis C in HIV-Coinfected Patients

**DOI:** 10.1371/journal.pone.0028115

**Published:** 2012-01-03

**Authors:** Luis F. López-Cortés, Rosa Ruiz-Valderas, Luis Jimenez-Jimenez, María F. González-Escribano, Almudena Torres-Cornejo, Rosario Mata, Antonio Rivero, Juan A. Pineda, Manuel Marquez-Solero, Pompeyo Viciana, on behalf of Grupo para el Estudio de las Hepatitis Víricas (HEPAVIR) de la Sociedad Andaluza de Enfermedades Infecciosas

**Affiliations:** 1 Unidad Clínica de Enfermedades Infecciosas, Hospitales Universitarios Virgen del Rocío/Instituto de Biomedicina de Sevilla, Seville. Spain; 2 Servicio de Bioquímica Clínica, Hospitales Universitarios Virgen del Rocio, Seville, Spain; 3 Servicio de Inmunología, Hospitales Universitarios Virgen del Rocío/Instituto de Biomedicina de Sevilla, Seville, Spain; 4 Unidad Clínica de Enfermedades Infecciosas, Hospital Universitario Reina Sofia, Córdoba, Spain; 5 Unidad Clínica de Enfermedades Infecciosas, Hospital Universitario de Valme, Seville, Spain; 6 Unidad Clínica de Enfermedades Infecciosas, Hospital Universitario Virgen de la Victoria, Málaga, Spain; Institut Pasteur, France

## Abstract

**Background:**

Data on which to base definitive recommendations on the doses and duration of therapy for genotype 3 HCV/HIV-coinfected patients are scarce. We evaluated the efficacy of a lower peginterferon-α 2a dose and a shorter duration of therapy than the current standard of care in genotype 3 HCV/HIV-coinfected patients.

**Methods and Findings:**

Pilot, open-label, single arm clinical trial which involved 58 Caucasian HCV/HIV-coinfected patients who received weekly 135 µg peginterferon-α 2a plus ribavirin 400 mg twice daily during 20 weeks after attaining undetectable viremia. The relationships between baseline patient-related variables, including IL28B genotype, plasma HCV-RNA, ribavirin dose/kg, peginterferon-α 2a and ribavirin levels with virological responses were analyzed.

Only 4 patients showed lack of response and 5 patients dropped out due to adverse events related to the study medication. Overall, sustained virologic response (SVR) rates were 58.3% by intention-to-treat and 71.4% by per protocol analysis, respectively. Among patients with rapid virologic response (RVR), SVR and relapses rates were 92.6% and 7.4%, respectively. No relationships were observed between viral responses and ribavirin dose/kg, peginterferon-α 2a concentrations, ribavirin levels or rs129679860 genotype.

**Conclusions:**

Weekly 135 µg pegIFN-α 2a could be as effective as the standard 180 µg dose, with a very low incidence of severe adverse events. A 24-week treatment duration appears to be appropriate in patients achieving RVR, but extending treatment up to just 20 weeks beyond negativization of viremia is associated with a high relapse rate in those patients not achieving RVR. There was no influence of IL28B genotype on the virological responses.

**Trial Registration:**

ClinicalTrials.gov NCT00553930

## Introduction

Although data on which to base definitive recommendations on the dosing and duration of therapy for co-infected patients are limited, the current standard therapy for chronic hepatitis C (CHC) genotype 3 (G3) is the combination of weekly 180 µg pegylated interferon-α (pegIFN-α) 2a or 1.5 µg/kg pegIFN-α 2b and 800 mg daily ribavirin (Rbv) for 48 weeks [1], [2]. This recommendation is based on a few randomized trials with both pegIFN-α 2a [3] and pegIFN-α 2b [4]–[7] and several cohort studies [8]–[10] in which sustained virologic response (SVR) rates between 34% and 82% were observed. In addition, as the HCV protease inhibitors are less active against G3 [11], the combination of pegIFN-α and Rbv will remain the key drugs for this genotype.

Besides, both pegIFN-α formulations are associated with large interpatient variability in plasma concentrations after specific doses [12], [13]. Previously, we evaluated the influence of pegIFN-α 2a plasma concentrations on the virologic response in HCV/HIV coinfected patients and, in contrast to G1/4, we found no association between pegIFN-α-2a levels and SVR in patients with G3 [14]. This fact, along with the similar SVR rate achieved for these G with lower doses of pegIFN-α 2a or 2b monotherapy [15]–[17], suggest that the pegIFN-α-2a levels achieved with the 180 µg weekly doses were in the plateau portion of the concentration–response curve for patients with G3.

On the other hand, the current standard treatment is associated with frequent and, sometimes, severe adverse events (AEs) and requires an extended duration of therapy. Therefore, a goal must be to find the minimal dose and duration that is effective for achieving SVR, decreasing the incidence or severity of AEs and enhancing adherence, particularly in HIV-coinfected patients in whom the treatment of HCV infection has been associated with a high rate of intolerance. In this study we evaluated the efficacy and safety of a lower pegIFN-α 2a dose (135 µg) plus flat dose ribavirin with shorter therapy duration (20 weeks after attaining undetectable serum HCV-RNA) in a cohort of HIV-coinfected patients with CHC G3. In this study we evaluated the efficacy and safety of a lower pegIFN-α 2a dose (135 µg) plus flat dose ribavirin with shorter therapy duration (20 weeks after attaining undetectable serum HCV-RNA) in a cohort of HIV-coinfected patients with CHC G3. After the study had begun, it was known that single nucleotide polymorphisms (SNPs) nearby the IL28B gene are strong predictors of response to pegIFN-α-2a plus Rbv treatment in CHC [18], [19]. So, we additionally assessed the influence of IL28B rs12979860 polymorphisms on the virological response to this regimen.

## Methods

### Ethics Statement

The protocol for this trial and supporting CONSORT checklist are available as supporting information; see Checklist S1 and Protocol S1. The study protocol was approved by the Agencia Española del Medicamento, and a central ethics committee (Comité Autonómico de Ensayos Clínicos, Consejería de Salud, Junta de Andalucía). The study was conducted according to the Declaration of Helsinki and current guidelines on Good Clinical Practices. All patients provided written informed consent. This study is registered at NIH register (ClinicalTrials.gov: NCT00553930) and EMEA (N° EudraCT: 2007-000814-35).

### Design and study population

This was a pilot, open-label, single arm, investigator-initiated clinical trial in which Caucasian HCV treatment naive patients were prospectively enrolled in four infectious disease units in Spain from January 2008 to August 2009. The trial ended after the completed follow-up of the last enrolled patient. Patients were eligible if they were older than 18 years, HIV-coinfected, and had CHC or compensated cirrhosis by HCV G3. Although the initial protocol contemplated the inclusion of patients carrying HCV G2, these were excluded from analysis as only two of these patients were enrolled. Patients were treated under routine clinical care conditions, and liver biopsy was not mandatory for inclusion in the study. Patients were excluded if they had active HIV-related opportunistic infection, cancer, hepatitis B coinfection, other causes of liver disease, hemoglobinopathies, previous history of severe psychiatric illness, autoimmune diseases, abnormal renal function, or pregnancy. There were no exclusion criteria regarding methadone use, CD4 cell counts, plasma HIV-RNA, or concurrent antiretroviral therapy.

### Laboratory tests

HCV genotypes were determined using a reverse hybridization assay (Inno-Lipa HCV II; Bayer, Barcelona, Spain). Plasma HCV-RNA loads were measured by a quantitative PCR assay (COBAS® AmpliPrep/COBAS® TaqMan® HCV Test; detection limit of 15 IU/ml) at baseline, after weeks 1, 2, 4, and monthly thereafter until the end of treatment, and again at 6 months after completing treatment. Hematological and biochemical profiles were also assessed at the same time points. CD4 counts (standard flow cytometry) and plasma HIV-RNA (Cobas AmpliPrep-Cobas TaqMan HIV-1 test, v.2.0, Basel, Switzerland) were measured at baseline and every three months thereafter. Hepatic fibrosis was evaluated by pretreatment liver biopsy according to the Scheuer's scoring system [20], or by transient elastography (Fibroscan®, Echosens) for which values of ≥11 and ≥14 kPa were considered as F3 and F4 fibrosis stages, respectively.

Genomic DNA was extracted from whole blood provided in EDTA tubes using the QIAmp DNA Blood Kit (Qiagen). The rs129679860 SNP was genotyped with a custom TaqMan genotyping assay (Applied Biosystems) on DNA isolated from whole blood samples. The DNA was genotyped according to the manufacturer's instructions on a MX3005 thermocycler using MXpro software (Stratagene).

Plasma samples for PegIFN-α-2a and Rbv C_min_ determinations were drawn on the morning of the day scheduled for weekly administration of pegIFN-α-2a after 11.45 to 12.15 hours to the previous Rbv dose (otherwise, blood samples were discarded) at weeks 1, 2, 4, and monthly afterwards until the end of therapy. Samples were stored at −80°C until tested. Plasma pegIFN-α-2a and Rbv concentrations were assayed by enzyme-linked immunosorbent assay Hu-INF-α (PBL Biomedical Laboratories, Piscataway, NJ), and reverse-phase high-performance liquid chromatography (HPLC-UV) as previously described [14]. All calibration standards, quality control samples, and study samples were analyzed in duplicate. Results are shown as the mean of the duplicates for the study samples.

### End-points and assessment of efficacy and safety

The primary efficacy end-point was SVR, defined as undetectable serum HCV-RNA at 24 weeks after completion of treatment. Efficacy data were assessed by both intention-to-treat (ITT) and by per-protocol analysis. Secondary end-points were to assess the influence of IL28B polymorphisms on SVR and the relationship between the plasma pegIFN-α-2a and Rbv concentrations and virological responses in patients receiving treatment.

Rapid virologic response (RVR) was defined as a plasma HCV-RNA<15 IU/mL at week 4 of treatment. Early virologic response (EVR) was defined as a plasma HCV-RNA<15 IU/mL or a decrease of ≥2 log_10_ IU/ml at week 12 of treatment or earlier, respectively. End of treatment response (ETR) was defined as undetectable serum HCV-RNA at the end of therapy. Relapses were defined as a detectable serum HCV-RNA at any time point after attaining ETR.

Safety was assessed by means of AEs reported by patients or detected by investigators, and laboratory results at scheduled visits on weeks 1, 2, 4, 8, 12, and monthly thereafter until the end of therapy, and categorized via a standardized toxicity grade scale (AIDS Clinical Trials Group). For toxicity and response investigations, analysis dropouts were considered until the last available visit.

### Drug treatment

Patients started treatment with the combination of weekly 135 µg pegIFN-α-2a plus oral Rbv (400 mg twice daily), and a planned duration of 20 weeks after attaining undetectable serum RNA-HCV. Treatment was discontinued at week 12 in patients not achieving EVR, and these cases were considered as virological failures. Antiretroviral treatment and use of erythropoietin remained at the responsible physicians' judgment.

### Statistical analysis

This pilot study was designed as a single-arm, uncontrolled trial, with a historical comparison. For ‘a priori’ sample size calculations, the Apricot trial, the largest clinical trial with pegIFN-α-2a plus Rbv conducted in HCV/HIV-coinfected patients (3,21) so far, was considered as historical reference. The SVR rate observed in patients harbouring HCV genotype 2 or 3 in the Apricot trial was 62%. Assuming a 57% SVR rate in our patients, a sample size of 60 patients would be required to reach a 80% statistical power with an asymptotic unilateral confidence interval of 95% and a 10% noninferiority margin (Ene 3.0; e-Biometria, Madrid, Spain).

Continuous variables are expressed as median (range) and categorical variables are expressed as the number of cases (percentage). For this analysis, the level of 800000 IU/mL was chosen as the cut-off point for plasma HCV-RNA concentration.

Intraindividual variability in plasma concentrations was assessed by measuring the coefficient of variation (CV = standard deviation/mean×100) of all the available values from each patient throughout the follow-up. Interindividual variability was calculated using the CV for the geometric mean of each subject. Ninety-five percent confidence intervals (CI_95_) around single proportions were calculated as *p*±1.96 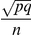 where p is the proportion and *q* = 1 − *p*. The relationships between virological responses and potentially predictive variables were examined using χ2 tests. To assess the independence of these factors, a logistic regression analysis was used in which all variables with P values below 0.2 in the initial analyses were included. All tests were two-tailed, and the differences were considered significant when P was<0.05. Statistical calculations were performed with the Statistical Product and Service Solutions for Windows (15.0 version, SPSS, Chicago, IL).

## Results

### Characteristics of the study population

A total of 60 Caucasian HCV/HIV-1-coinfected patients (G2, 2; G3, 58) were included in the study in four infectious disease units in Spain from January 2008 to August 2009. The trial ended after the completed follow-up of the last enrolled patient. The two patients with HCV G2 were excluded from analysis for homogenizing results. Baseline characteristics are summarized in table 1. Information on liver fibrosis was available for 56 patients (by liver biopsy in 23, by transient elastography in 41, and by both techniques in 16); 21 (36.2%) of these 58 patients had cirrhosis. Baseline HCV-RNA was similar in patients with CC rs129679860 IL28B genotype (5.93 log_10_ IU/ml; range, 3.02 – 7.15) and in those carrying the T allele (5.55; range, 3.52 – 7.13; p = 0.4).

**Table 1:** Baseline characteristics of the patients included in the study.

Male, no. (%)	48 (82.8)
Age, years, M (range)	44 (27 – 57)
Weight, kg, M (range)	69 (41.3 – 102.0)
Risk factor for HIV-HCV, no. (%)	
Previous injection drug use	47 (81.0)
Other	11 (18.9)
Previous C stage (CDC), no. (%)	10 (17.2)
CD4/µl, M (range)	395 (92 – 1500)
<200/µl, no. (%)	3 (5.2)
HIV-RNA copies/mL, M (range)	<50 (<50 – 64200)
>50 copies/mL, no. (%)	13 (22.4)
Antiretroviral treatment, no. (%)	51 (87.9)
2 N(t)RTIs+1 PI	29 (50.0)
2 N(t)RTIs+1 NNRTI	16 (27.6)
Other	6 (10.3)
HCV RNA IU/ml, M (range)	530975 (1054 – 14085574)
>800000 IU/ml, no. (%)	21 (36.2)
Elevated ALT, no. (%)	48 (82.7)
LDL-C mg/dL, M (range)	84 (27 – 181)
Fibrosis stage (biopsy or FibroScan), no. (%)	56 (96.6)
F 0–1	26 (46.4)
F 2	6 (10.3)
F 3	3 (5.2)
F 4	21 (36.2)
IL-28B (rs129679860 SNP), no. (%)	47 (78.3)
CC	25 (43.1)
CT	19 (32.8)
TT	1 (1.7)

M: median. N(t)RTIs: nucleos(t)ide analogue reverse transcriptase inhibitors. NNRTI: nonnucleoside reverse transcriptase inhibitor. PI: protease inhibitor. LDL-C: low-density lipoprotein cholesterol.

### Treatment efficacy and safety

**Figure 1:**
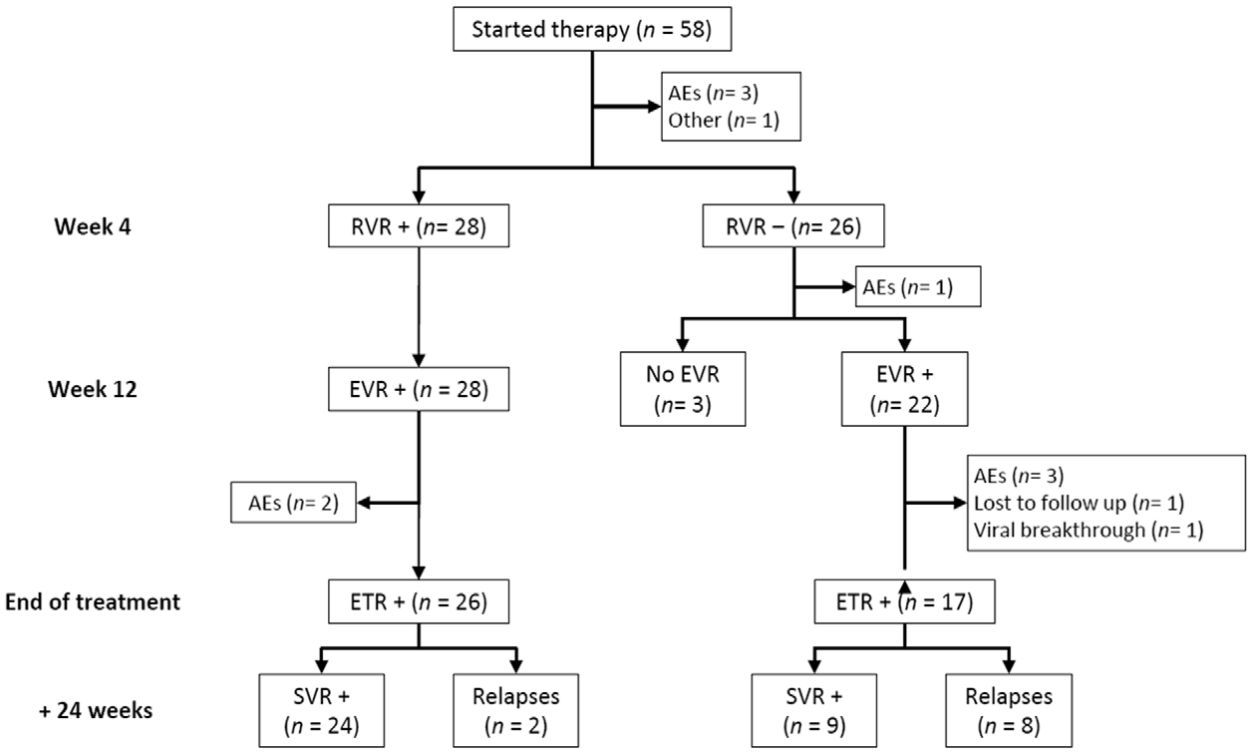
Study flow diagram. RVR: rapid virological response. EVR: early virological response. ETR: end of treatment response. SVR: sustained virological response. AEs: adverse events.

The study flow diagram and virological responses are shown in figure 1, and table 2, respectively. Fifteen patients (25.8%) prematurely discontinued treatment, 4 (6.8%) due to lack of response and 11 due mainly to AEs. Although 55 patients (94.8%) presented one or more AEs (table 3), especially within the first 12 weeks of therapy, the AEs considered related to the study medication led to discontinuation of treatment in only 4 (6.9%) patients (nausea and vomiting, 2; depression, 1; pneumococcal pneumonia, 1). In 5 additional patients the AEs were considered unrelated to the study medication. Ten of these latter patients could be considered as responders as 4 of them discontinued therapy with a plasma HCV-RNA reductions of 2.59 to 3.65 after 1 or 2 weeks of therapy and 6 of them 7 remaining patients did it after reaching a negative viremia at week 8 or 12. Finally, 43 patients completed the treatment (figure 1) with a median duration of 24 weeks (range, 24 – 42). Only 12 patients extended treatment beyond 24 weeks (28, 6; 32, 2; 36, 3, and 42, 1), and 3 patients had a treatment exposition less than 80% of the planned treatment. There were no dose reductions of pegIFN-α-2a or Rbv, and only 2 patients required erythropoietin (30000 IU weekly) from weeks 4 and 20 onwards, respectively.

**Table 2:** Viral responses in HCV/HIV-coinfected patients treated with pegIFN α-2a (135 µg/week) plus ribavirin (800 mg/day).

	RVR	EVR	ETR	SVR	Relapse
**ITT analysis** (n = 58), no. (%)	28 (48.3)	50 (86.2)	43 (74.1)	33 (56.9)	10/43 (23.2)
CI_95_ (%)	(35.5–61.1)	(77.4–95.0)	(62.5–85.7)	(43.8–70.0)	(10.1–36.4)
≤800000 IU/mL (n = 37), no. (%)	23 (62.2)	33 (89.2)	27 (73)	24 (64.9)	3/27 (11.1)
>800000 IU/mL (n = 21), no. (%)	5 (23.8)	17 (81.0)	16 (76.2)	9 (42.9)	7/16 (43.8)
	n = 54	n = 53	n = 47	n = 47	n = 43
**Per-protocol analysis**, no. (%)	28 (51.9)	50 (94.3)	43 (91.4)	33 (70.2)	10 (23.2)
CI_95_ (%)	(38.1–65.6)	(87.9–100)	(83.2–100)	(56.6–83.8)	(10.1–36.4)
≤800000 IU/mL, no. (%)	23 (67.6)	33 (97.1)	27 (93.1)	24 (82.8)	3 (11.1)
	n = 34	n = 34	n = 29	n = 29	n = 27
>800000 IU/mL, no. (%)	5 (25.0)	17 (89.5)	16 (88.9)	9 (50.0)	7 (43.8)
	n = 20	n = 19	n = 18	n = 18	n = 16

ITT: intention to treat. RVR: rapid virological response. EVR: early virological response. ETR: end of treatment response. SVR: sustained virological response.

**Table 3:** Adverse events or intercurrent illness throughout treatment with weekly pegIFN-α-2a (135 µg) plus oral Ribavirin (400 mg twice daily); n = 58 patients.

	Total	Grade 1 -2	Grade 3	Grade 4
	no. (%)	no. (%)	no. (%)	no. (%)
Flu-like syndrome	30 (51.7)	27 (46.5)	3 (5.1)	
Myalgia	9 (15.5)	8 (13.7)	1 (1.7)	
Fatigue	28 (48.2)	28 (48.2)		
Unintentional weight loss ≥10%	14 (24.1)		14 (24.1)	
Anorexia	23 (39.6)	23 (39.6)		
Depression	14 (24.1)	13 (22.4)	1 (1.7)	
Irritability - nervousness	12 (20.6)	11 (18.9)	1 (1.7)	
Insomnia	11 (18.9)	10 (16.7)	1 (1.7)	
Nausea – vomiting	15 (25.8)	15 (25.8)		
Anemia	7 (12.0)	6 (10.3)	1 (1.7)	
Neutropenia	21 (36.2)	18 (31.0)	3 (5.1)	
Thrombocytopenia	10 (18.3)	10 (18.3)		
Other				
- Subarachnoid hemorrhage (death)	1 (1.7)			1 (1.7)
- Neuroleptic malignant syndrome (death)	1 (1.7)			1 (1.7)
- Sudden death	1 (1.7)			1 (1.7)
- Pneumococcal pneumonia	1 (1.7)			1 (1.7)
- Esophageal stenosis (relapse)	1 (1.7)	1 (1.7)		
- Thrush	1 (1.7)	1 (1.7)		
- Deep vein thrombosis	1 (1.7)	1 (1.7)		
- Cough	2 (3.4)	2 (3.4)		
- Headache	2 (3.4)	2 (3.4)		
- Dizziness	1 (1.7)	1 (1.7)		
- Injection site erythema	1 (1.7)	1 (1.7)		

Overall, SVR rates were 56.9% (CI_95_: 43.8 – 70.0%) by ITT, and 70.2% (CI_95_: 56.6 – 83.8%) by per protocol analysis, with a relapse rate of 23.2% (CI_95_: 10.1 – 36.4%). Among those 28 patients who had RVR, 24 (85.7%) attained SVR by ITT analysis, with rates of SVR and relapses of 92.3% and 7.7%, respectively, by per-protocol analysis. On the other hand, among the 32 patients who did not achieve RVR, 9 of them (28.1%) attained SVR and 8 relapsed.

### Pharmacokinetics results for pegIFN-α-2a and Rbv

**Figure 2:**
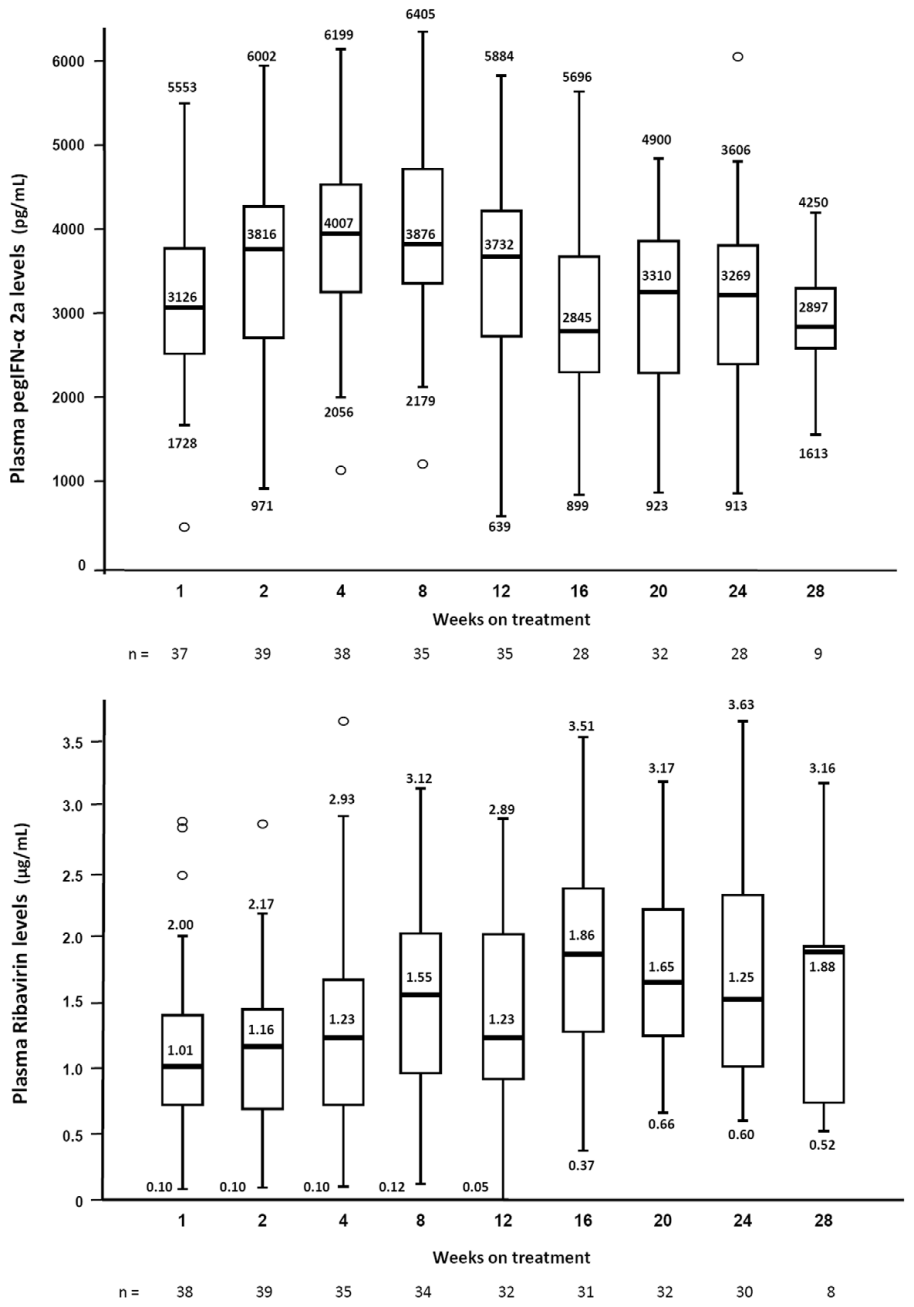
Peginterferon-α-2a (A) and ribavirin (B) trough plasma levels at different time points. Median (range).

A total of 281 samples were available for pegIFN-α-2a and Rbv determinations throughout the follow-up period, with a median of 6 samples per patient (range, 1 – 9 for pegIFN-α-2a, and 2 -10, for Rbv). The median Rbv dose was 11.6 mg/kg (range, 7.8 – 19.4). The results of drug plasma levels are shown in figure 2. Median intrapatient CV for PegIFN-α-2a and Rbv levels were 24.6% (range, 0.08 – 0.92) and 40.7% (range, 0.8 – 1.37), and interpatient variation coefficients were 58.9% and 56.5%, respectively. No significant differences were found in pegIFN-α-2a concentrations regarding gender, weight or the presence of cirrhosis.

No relationship was observed between Rbv levels and body weight (r = −0.35; P = 0.8), the administered dose/kg (r = 0.012; P = 0.9) or the estimated glomerular filtration rate (r = −0.88; P = 0.5).

### Variables associated with viral responses and relapses

**Figure 3:**
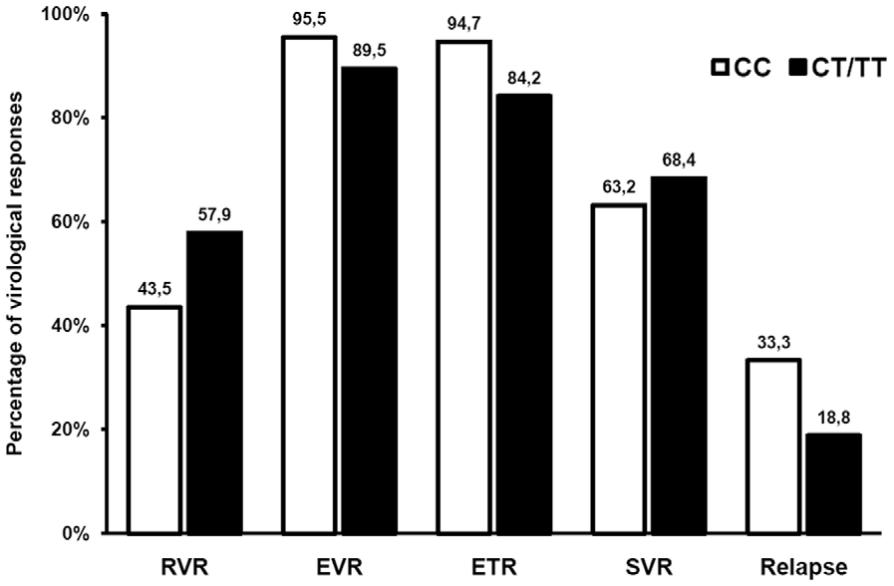
Percentage of virological responses and relapses as function of rs129679860 genotype (CC vs. CT/TT). RVR: rapid virological response; EVR: early virological response; ETR: end of treatment response; SVR: sustained virological response.

The relationships between baseline patient-related variables including IL-28B genotype, plasma HCV-RNA, Rbv dose/kg, PegIFN-α 2a and Rbv levels with RVR, EVR, ETR, SVR and relapses are shown in table 4. In the multivariable analysis only HCV-RNA<800000 UI/mL remained independently associated with RVR (OR: 5.36; CI_95_: 1.04 – 27.81; P = 0.04), SVR (OR: 6.68; CI_95_: 1.02 – 47.08; P = 0.05), and relapses (OR: 0.14: CI_95_: 0.021 – 0.99; P = 0.05). Specifically, no relationships were observed between any virological responses or relapses and rs129679860 genotype (figure 3), Rbv dose/kg, PegIFN-α-2a or Rbv levels.

**Table 4:** Factors predictive of rapid (RVR) and sustained virological response (SVR) to anti-hepatitis C virus therapy.

	Univariate nonparametric analysis				Multivariable analysis			
					RVR		SVR	
Factor	RVR (%)	*P*	SVR (%)	*P*	Adjusted OR (CI_95_)	*P*	Adjusted OR (CI_95_)	*P*
**Plasma HCV RNA**								
(≤vs.>800000 IU/mL)	67.6 vs. 25.0	0.003	82.8 vs. 50.0	0.02	5.36 (1.04 – 27.81)	0.04	6.68 (1.01 – 47.08)	0.05
**LDL-C mg/dL**								
(≥80 vs.<80)	70.0 vs. 33.3	0.02	88.9 vs. 47.4	0.008	3.08 (0.67 – 14.05)	0.14	3.31 (0.45 – 24.33)	0.23
**Cirrhosis**								
(no vs. yes)	60.6 vs. 40.0	0.12	74.2 vs. 62.5	0.30	2.51 (0.51 – 12.23)	0.25		
**Use of TDF vs. ABV**	54.5 vs. 60.0	0.49	72.4 vs. 87.5	0.35	n.a.		n.a.	
**IL28B**								
(CC vs. CT/TT)	43.5 vs. 42.1	0.26	63.2 vs. 68.4	0.50	n.a.		n.a.	
**Treatment exposition**								
(≥80% vs.<80%)	n.a.		81.6 vs. 40.0	0.07	n.a.		7.81 (0.37 – 161.4)	0.18

TDF: tenofovir. ABV: abacavir. n.a.: not applicable.

Receiver-operating characteristic curve analysis showed that the ability of PegIFN-α 2a and Rbv plasma concentrations to identify viral responders was null. Particularly, the areas under the curve for RVR, EVR, ETR and SVR for PegIFN-α 2a levels were 0.505 (CI_95_, 0.315 – 0.697; p = 0.9), 0.811 (CI_95_, 0.539 – 1; p = 0.07), 0.697 (CI_95_, 0.338 – 1; p = 0.2), and 0.587 (CI_95_, 0.372 – 0.801; p = 0.4), respectively. For Rbv these values were 0.673 (CI_95_, 0.509 – 0.839; p = 0.4), 0.380 (CI_95_, −0.028 – 0.784; p = 0.3), 0.375 (CI_95_, 0.040 – 0.703; p = 0.4), and 0.632 (CI_95_, 0.043 – 0.817; p = 0.2), respectively. Nevertheless, the regression model based on the selected variables explained only moderately the observed variability in the SVR as Nagelkerke's R^2^ was 0.439.

## Discussion

This pilot study was aimed to evaluate two hypothesis; first, the viral efficacy of a pegIFN-α-2a dose lower than the standard of care, and, second, if a treatment duration of 20 weeks after attaining undetectable serum HCV-RNA was sufficient in G 3 HCV/HIV-coinfected patients. Regarding the first hypothesis, the dose-ranging studies with both formulations of PegIFN-α showed that lower than the standard doses (90 or 135 µg weekly for pegIFN-α 2a, and 0.75 µg/kg weekly for pegIFN-α 2b) achieved similar SVR rates in HCV monoinfected patients with G 3, both as a single agent [15]–[17] and in combination with Rbv [22]–[24]. However, to date there are no data available, to our knowledge, on the use of low doses of PegIFN-α in HCV/HIV coinfected patients.

Our results suggest that pegIFN-α-2a given at 135 µg once weekly might be as effective as the standard 180 µg dose, when administered together with 800 mg daily of Rbv in G3 HCV/HIV-coinfected patients, since both on treatment RVR and EVR rates in our study (51.9%, and 94.3%, respectively) were similar to those observed in other studies as the Apricot trial (37%, and 88%, respectively) [21], and our previous study (RVR, 58.3%; EVR, 97.5%) in which similar patients were treated with weekly 180 µg pegIFN-α 2a [14]. These results are despite the use of a stricter criterion for a negative HCV viremia in the current study (≤15 UI/mL by quantitative PCR assay vs.<50 UI/mL by qualitative PCR). In fact, five patients in our study had viremia levels between 18 and 48 IU/mL at week 4, which would have rated them as rapid responders by qualitative methods. Unfortunately, there are no other studies on pegIFN-α 2a plus Rbv (800 mg/day) in HIV-coinfected patients with CHC G3 which reported EVR rates on treatment for comparison. In the Presco study a SVR rate of 79.3% was observed after standard pegIFN-α-2a 180 µg dose plus higher Rbv dose (1000–1200 mg/day) instead a flat dose of 800 mg/day [25].

Our hypothesis is also supported by the lack of relationship between pegIFN-α 2a plasma levels and the rate of viral response, despite those being lower than that observed previously with pegIFN-α 2a at 180 µg once weekly (2467; range, 757 – 5136 vs. 4762 pg/mL; range, 1419 – 6451) [14]. However, while a treatment duration of 20 weeks after attaining undetectable serum HCV-RNA seems adequate in patients achieving RVR regardless of their baseline HCV-RNA (in fact, both relapsing patients had baseline viremias<5000 UI/mL), the high relapse rate in patients with no RVR suggests that extending treatment to only 20 weeks after achieving negativization of viremia is not sufficient and thus a longer treatment period is needed.

No relationship was observed between Rbv plasma levels and viral responses during therapy or SVR rate, similar to results in our previous study [14]. Moreover, we did not find any relationship between the administered dose per kg of Rbv and its plasma levels achieved at just 12 hours after drug intake. Therefore, we believe the current recommendation of dosing Rbv in mg/kg according to the patient's weight is still a matter of debate, particularly in G2/3 in which doses of 400 and 800 mg/day might produce equivalent SVR rates in patients infected with HCV G3 [26].

Likewise, we observed no relationship between rs129679860 IL28B genetic polymorphisms and virological responses to pegIFN-α 2a plus Rbv in our patients with CHC G3, as has been previously reported in both G3 monoinfected and HCV/HIV-coinfected patients [27]–[29], although the existing data on this issue are not entirely homogeneous [30].

Regarding the incidence of toxicity, it is remarkable that both clinical and laboratory AEs were just mild and did not motivate dose reductions in patients receiving the 135 µg dose. Four out of the 8 cases of AEs which motivated treatment interruption were considered unrelated to study treatment. Thus, the rate of drop-outs due to treatment-related toxicity was only 6.9%, which compare favorably with previous studies in which the rate of serious AEs varied between 10 and 18% [4], [5], [9], [11]. Moreover, these drop-outs were mainly caused by poor tolerance rather than by the severity of the AEs.

The main limitation of our study is the fact that it is a single-arm trial in which the results were compared with those observed in earlier clinical trials. However, we believe such a study had to be carried out prior to the design of a randomized controlled trial allowing for a one-to-one comparison between efficacy and safety of weekly 135 and 180 µg doses of pegIFN-α-2a in G3 HCV/HIV-coinfected patients. Furthermore, a high drop-out rate (18.9%), mainly due to low grade AEs and those unrelated to the study medication, penalized the intention-to-treat analysis results.

In conclusion, our results suggest that weekly 135 µg pegIFN-α 2a could be as effective as the standard 180 µg dose, with lower incidence of severe AEs, supporting the design of a randomized controlled trial allowing for a one-to-one comparison between efficacy and safety of weekly 135 and 180 µg doses of pegIFN-α-2a in G3 HCV/HIV-coinfected patients. Our data evidence that neither the rs129679860 IL-28B genetic polymorphisms nor plasma pegIFN-α-2a or Rbv levels influence the virological responses in G3 HCV/HIV-coinfected patients. On the other hand, while a 24-week treatment duration appears to be appropriate in patients achieving negative viremia on week 4, extending treatment duration up to just 20 weeks beyond negativization of viremia is associated with a high relapse rate in those patients not achieving RVR, and therefore a more prolonged treatment is warranted in these patients.

## Supporting Information

Checklist S1 **CONSORT Checklist.**
(DOC)

Protocol S1 **Trial protocol.**
(PDF)
