# Supplementary material for: Influence of IL28B Polymorphisms on Response to a Lower-Than-Standard Dose peg-IFN-α 2a for Genotype 3 Chronic Hepatitis C in HIV-Coinfected Patients
Source: PLoS One. 2012 Jan 3;7(1):e28115. doi: 10.1371/journal.pone.0028115 (PMC3250391; doi:10.1371/journal.pone.0028115)
Supplement: Protocol S1 — Trial protocol. (PDF) [file pone.0028115.s002.pdf]

Fecha:* *04/12/2007

***Referencia:* MUH/CLIN**

RESOLUCIÓN DE AUTORIZACIÓN DEL ENSAYO CLINICO Nº EudraCT 2007-000814-35

DESTINATARIO: **D. Luis Fernando López Cortés**
Avda. Manuel Siurot, s/n
41013 - Sevilla
Sevilla
España

Vista la solicitud formulada por **Dr. Luis F. Lopez Cortes** para la realización del ensayo clínico número EudraCT **2007-000814-35** titulado **"Eficacia de dosis bajas de interferón pegilado alfa-2a junto con ribavirina en el tratamiento de la hepatopatía crónica por virus C (genotipos 2 o 3) en pacientes coinfectados por el VIH"**, código de protocolo del promotor **SAEI_IFN_1**, cuyo promotor es **D. Luis Fernando López Cortés** se emite resolución a tenor de los siguientes

ANTECEDENTES DE HECHO

PRIMERO: Con fecha 06/09/2007 presenta solicitud de autorización de ensayo clínico.

SEGUNDO: Con fecha 08/11/2007 se han solicitado aclaraciones que han sido respondidas de forma satisfactoria con fecha 15/11/2007.

A estos antecedentes de hechos le son de aplicación los siguientes:

FUNDAMENTOS DE DERECHO:

Único. Son de aplicación al presente procedimiento la Ley 30/1992, de 26 de noviembre, de Régimen Jurídico de las Administraciones Públicas y del Procedimiento Administrativo Común, modificada por la Ley 4/1999, de 13 de enero; la Ley 12/2000, de 29 de diciembre de medidas fiscales, administrativas y de orden social; Ley 29/2006, de 26 de julio, de Garantías y Uso Racional de Medicamentos y Productos Sanitarios; el Real Decreto 223/2004 de 6 de febrero, por el que se regulan los ensayos clínicos con medicamentos; el Real Decreto 520/1999, de 26 de marzo, por el que se aprueba el Estatuto de la Agencia Española del Medicamento, y demás normas aplicables,

Así, del expediente se deduce que se cumplen los requisitos establecidos para su autorización de acuerdo con el Articulo 22 del Real Decreto 223/2004.

Por todo lo anteriormente expuesto la Directora de la Agencia de Medicamentos y Productos Sanitarios en el ejercicio de sus competencias **RESUELVE:**

1º.- AUTORIZAR la realización de este ensayo clínico número EudraCT 2007-000814-35,

Contra esta Resolución, que pone fin a la vía administrativa, puede interponerse potestativamente Recurso de Reposición ante el/la Director/a de la Agencia Española de Medicamentos y Productos Sanitarios en el plazo de un mes, conforme a lo dispuesto en el artículo 116 de la Ley 30/1992, de 26 de noviembre, de Régimen Jurídico de las Administraciones públicas y del Procedimiento Administrativo Común, o interponerse Recurso Contencioso-Administrativo ante el Juzgado Central de lo Contencioso-Administrativo de Madrid, en el plazo de dos meses a contar desde el día siguiente a la recepción de la presente notificación, conforme a lo dispuesto en la Ley Reguladora de la Jurisdicción Contencioso-Administrativa de 13 de julio de 1998, y sin perjuicio de cualquier otro recurso que pudiera interponerse.

LA DIRECTORA DE LA AGENCIA ESPAÑOLA
DE MEDICAMENTOS Y PRODUCTOS SANITARIOS

agencia española de medicamentos y productos sanitarios
Ministerio de Sanidad y Consumo

Dª. Cristina Avendaño Solá

ANTONIO VELÁZQUEZ MARTÍNEZ, COMO SECRETARIO DEL **COMITÉ AUTONÓMICO DE ENSAYOS CLINICOS DE ANDALUCÍA**

CERTIFICA

Que este Comité ha evaluado en Andalucía la propuesta del promotor Dr. Luis Fernando López Cortés (Hosp. Univ. Virgen del Rocío), para que se realice el Ensayo Clínico código de Protocolo del Promotor SAEI_IFN_1, perteneciente a la convocatoria del Instituto de salud Carlos III (BOE num 28, de 1 de febrero de 2007), y num. De EudraCT: 2007- 000814-35 titulado:

Eficacia de dosis bajas de interferón pegilado alfa-2a junto con ribavirina en el tratamiento de la hepatopatía crónica por virus C (genotipos 2 ó 3) en pacientes coinfectados por el VIH
Protocolo, Hoja de Información al Paciente y Consentimiento Informado versión 1 de 11/02/07

con el medicamento **pegilado alfa-2a y ribavirina** y considera que:

El ensayo se plantea siguiendo los requisitos del Real Decreto 223/2004, de 6 de febrero y las normas que lo desarrollan y su realización es pertinente.

Se cumplen los requisitos necesarios de idoneidad del protocolo en relación con los objetivos del estudio y están justificados los riesgos y molestias previsibles para el sujeto, teniendo en cuenta los beneficios esperados.

El seguro o la garantía financiera previstos son adecuados.

El procedimiento para obtener el consentimiento informado y el plan de reclutamiento de sujetos previstos son adecuados, así como las compensaciones previstas para los sujetos por daños que pudieran derivarse de su participación en el ensayo.

La capacidad del investigador y sus colaboradores y las instalaciones y medios disponibles son apropiados para llevar a cabo el estudio.

El alcance de las compensaciones económicas previstas no interfiere con el respeto a los postulados éticos.

Por tanto, este Comité Autonómico como **comité de referencia**, habiendo tenido en cuenta los dictámenes de todos los CEICs implicados acepta que dicho ensayo clínico sea realizado en los centros siguientes por los investigadores principales que se relacionan a continuación

Centro	Investigador
Hosp. Univ. Virgen del Rocío (Sevilla)	Dr. Luis Fernando López Cortés
Hosp.. Reina Sofía (Córdoba)	Dr. Antonio Rivero Román
Hosp.. Virgen de Valme (Sevilla)	Dr. Ángel García García
Hosp.. Virgen de la Victoria (Málaga)	Dra. Mercedes González Serrano

Lo que firmo en **Sevilla**, a **veintinueve de marzo de dos mil siete**

JUNTA DE ANDALUCIA
Secretaría General de Calidad y Modernización
Comité Autonómico de Ensayos Clínicos
SEVILLA

ANTONIO VELÁZQUEZ MARTÍNEZ, COMO SECRETARIO DEL COMITÉ AUTONÓMICO DE ENSAYOS CLINICOS DE ANDALUCÍA

CERTIFICA

Que este Comité ha ponderado y evaluado en sesión celebrada el veintisiete de marzo de dos mil siete (Acta 03/07), y a propuesta del promotor Dr. Luis Fernando López Cortés (Hosp.. Univ. Virgen del Rocío), para que se realice el Ensayo Clínico código de Protocolo del Promotor SAEI_IFN_1, perteneciente a la convocatoria del Instituto de salud Carlos III (BOE num 28, de 1 de febrero de 2007), y num. De EudraCT: 2007-000814-35 titulado:

Eficacia de dosis bajas de interferón pegilado alfa-2a junto con ribavirina en el tratamiento de la hepatopatía crónica por virus C (genotipos 2 ó 3) en pacientes coinfectados por el VIH

Protocolo, Hoja de Información al Paciente y Consentimiento Informado versión 1 de 11/02/07

Que a dicha sesión asistieron los siguientes integrantes del Comité:

Sr. D. Mariano Aguayo Canela (Presidente. Medicina Interna. Hosp. Univ. Virgen Macarena. Sevilla)
Sr. D. Antonio Velázquez Martínez (Secretario. Medicina Preventiva y Salud P. Consejería de Salud)
Sr. D. Fernando Antúnez Estévez (Vocal. Médico- Lcdo. Derecho. Delegación Prov. de Salud. Granada)
Sra. Dª Encarnación Blanco Reina (Vocal. Farmacóloga Clínica. Universidad de Málaga)
Sr. D. Francisco Javier Bautista Paloma (Vocal. Farmacia Hospitalaria. H. U. Virgen del Rocío. Sevilla)
Sr. D. Juan Ramón Castillo Ferrando (Vocal. Farmacología Clínica. H. U. Virgen del Rocío. Sevilla)
Sra. Dª. Lourdes de la Colina Fernández (Vocal. Psiquiatría. Hosp. Juan Ramón Jiménez. Huelva)
Sr. D. Miguel Delgado Rodríguez (Vocal. Ajeno a la Institución. Univ. de Jaén, Médico Med. Preventiva)
Sr. D. Juan Carlos Domínguez Camacho (Vocal. Farmacéutico. Distrito Sanitario Sevilla)
Sra. Dª. María Ángeles López de la Torre (Vocal. Lcdo. en Derecho. H. U. Virgen del Rocío. Sevilla)
Sra. Dª Salud Luna Lagares (Vocal. Pediatría. Hosp. de Jerez)
Sr. D. Manuel de la Mata García (Vocal. Ap. Digestivo. Hosp. Univ. Reina Sofía. Córdoba)
Sr. D. José Miguel Morales Asencio (Vocal. Enfermero. D.S. Málaga. Málaga)
Sr. D. Emilio Perea –Milla López (Vocal. Epidemiólogo. Hosp.. Costa del Sol. Málaga)
Sr. Dª. María Ángeles Rasero Díaz (Vocal. Ajeno a las profesiones sanitarias. Consejería de Salud)
Sra. Dª Susana Rodríguez Domínguez (Vocal. Médico de Familia. D.S. Sevilla. Sevilla)

Que dicho Comité, está constituido y actúa de acuerdo con la normativa vigente y las directrices de la Conferencia Internacional de Buena Práctica Clínica.

Lo que firmo en **Sevilla**, a **veintinueve de marzo de dos mil siete**

Avda. de la Innovación, s/n. Edificio Arena 1. Apdo. Correos 17.111. 41080 Sevilla
Teléf. 95 500 65 56. Fax 95 500 66 84

ClinicalTrials.gov
Protocol Registration System

Protocol Registration Receipt
2007-11-03

Low Dose Peginterferon-α 2a for Chronic Hepatitis C, Genotypes 2 or 3, in HIV-Coinfected Patients (SAEI_IFN_1)

This study is currently recruiting participants.

Verified by Sociedad Andaluza de Enfermedades Infecciosas 2007-11

Sponsors and Collaborators:	Sociedad Andaluza de Enfermedades Infecciosas
Information provided by:	Sociedad Andaluza de Enfermedades Infecciosas
ClinicalTrials.gov Identifier:	

Purpose

Hypothesis: A regimen of low dose of peginterferon alfa-2a plus ribavirin may be as effective as currently recommended regimen for chronic hepatitis C in HIV-coinfected patients.

Objective: To evaluate the efficacy of lower dose of pegylated interferon-α 2a (135 µg weekly) plus ribavirin and a shorter duration of treatment (20 weeks after achieving an undetectable plasmatic HCV-RNA)than the current recommended in patients with chronic hepatitis or compensated cirrhosis by hepatitis C virus, genotypes 2 or 3, in HIV-coinfected patients in real use conditions.

Method: Phase IV, postautorization, open labelled multicenter trial with a planned duration of 118 weeks in which 71 patients from several hospitals of the Servicio Andaluz de Salud will be enrolled. The usual clinical and analytical follow up will be performed but additional blood samples will be obtained for determination of interferon and ribavirin plasma levels. The primary end point wall be a sustained virologic response (defined as an undetectable serum HCV-RNA after 24 weeks after the cessation of treatment). Likewise, rapid virological response (at 4 weeks of treatment), early virological response (at 12 weeks), and end of treatment response rates will be evaluated as well as their relationships with the plasma interferon an ribavirin concentrations determined by ELISA and HPLC, respectively. The safety and tolerability of the studied

medications will be evaluated by means of clinical adverse events, physical examination and laboratory results.

Condition	Intervention	Phase
Hepatitis C, Chronic	Drug: Pegylated interferon alfa-2a and Ribavirin<br>Drug: Pegylated interferon alfa 2a and Ribavirin	Phase 4

Study Type: Interventional
Study Design: Treatment, Non-Randomized, Open Label, Historical Control, Single Group Assignment, Pharmacokinetics/Dynamics Study

Number of arms in study: 1

Official Title: Efficacy of Low Dose Pegylated Interferon-α 2a Plus Ribavirin for the Treatment of Chronic Hepatitis C, Genotypes 2 or 3, in HIV-Coinfected Patients.

Primary Outcomes:

- Sustained viral response(undetectable serum HCV-RNA) [Time Frame: 24 weeks after the cessation of treatment]

Secondary Outcomes:

- Relationships between the plasma interferon an ribavirin concentrations and efficacy. The safety and tolerability of the studied medications will be evaluated by means of clinical adverse events, physical examination and laboratory results. [Time Frame: Throughout treatment and 24 weeks after finishing it]

Further Study Details:

Expected Total Enrollment: 71
Study Start: 2007-11; Expected Completion: 2009-12

Arms	Assigned Interventions
G 2/3: Experimental<br>Patients with chronic hepatitis or compensated cirrhosis by hepatitis C virus, genotypes 2 or 3, and HIV-coinfected.	Drug: Pegylated interferon alfa-2a and Ribavirin<br>All patients will be treated with the combination of pegIFN-α 2a (135 μg per week)plus oral Ribavirin at a dose of 800 mg

Arms	Assigned Interventions
	per day. The treatment will be continued up to 20 weeks after reaching an undetectable plasma RNA-HCV. Treatment will be discontinued for patients who did not achieve a reduction of al least 2 log10 IU/ml in plasma HCV RNA levels with respect to baseline at week 12 and will be considered as viral failures.<br><br>Drug: Pegylated interferon alfa 2a and Ribavirin<br>Pegylated interferon alfa 2a (135 ug/week)and Ribavirin (800 mg/day). Duration: 20 weeks after reaching an undetectable plasma RNA_HCV. Treatment will be discontinued for patients who did not achieve a decrease of >= 2 log10 IU/ml in plasma HCV RNA levels with respect to baseline at week 12 of treatment or earlier and will be considered as viral failures.

Eligibility

Ages Eligible for Study: 18 Years and above , Genders Eligible for Study: Both

Criteria

Inclusion Criteria:

- Age older than 18 years
- HIV infected, diagnosed with chronic hepatitis or compensated cirrhosis by hepatitis C virus (both anti-HCV antibodies and HCV RNA levels detectable in serum) not previously treated.
- Women of child-bearing age: negative pregnancy test
- Ability to understand and sign a written consent form

Exclusion Criteria:

- Previous interferon treatment
- Pregnancy or breastfeeding
- Acute or chronic hepatitis B infection (positivity for hepatitis B surface antigen or plasma DNA)
- Creatinine clearance < 50 ml/min, according to Cockcroft-Gault
- Decompensated liver disease
- History of organ transplantation
- Concomitant treatment with immunomodulators or didanosine
- Alcohol abuse or use of other recreational drugs
- History of severe psychiatric conditions
- Autoimmune diseases
- Inability to understand and sign a written consent form

Location and Contact Information

Luis F Lopez-Cortes, MD, PhD 34 - 955013096 lflopez@telefonica.net

Luis F Lopez-Cortes, MD, PhD 34 - 955013096 lflopez@telefonica.net

Spain, Sevilla

Infectious Diseases Service.Hospitales Universitarios Virgen del Rocio, Sevilla, Sevilla, 41013, Spain; Recruiting

Luis F Lopez-Cortes, MD, PhD, Principal Investigator

Study chairs or principal investigators

Luis F Lopez-Cortes, MD, PhD, Study Director

Infectious Disease Service. Hospitales Universitarios Virgen del Rocio. Sevilla. Spain

Antonio Rivero, MD, PhD, Principal Investigator

Hospital Universitario Reina Sofía. Córdoba. Spain

Mercedes Gonzalez, MD, PhD, Principal Investigator

Hospital Universitario Virgen de la Victoria. Malaga. Spain

Angel Garcia, MD, Principal Investigator

Hospital Universitario de Valme. Sevilla. Spain

More Information

Study ID Numbers SAEI_IFN_1

Health Authority: Spain: Spanish Agency of Medicines

PROTOCOLO DE ENSAYO CLINICO

Eficacia de dosis bajas de interferón pegilado alfa-2a junto con ribavirina en el tratamiento de la hepatopatía crónica por virus C (genotipos 2 o 3) en pacientes coinfectados por el VIH

Código del protocolo: SAEI_IFN_1

Nº EudraCT: 2007-000814-35

Versión 13-11-2007

Declaración de confidencialidad

La información contenida en este documento, especialmente los datos no publicados, es propiedad del Promotor (o está bajo su control) y, por consiguiente, se le proporciona de manera confidencial en calidad de investigador, posible investigador o consultor, para que sea revisada por usted, su equipo, Los Comités Éticos de Investigación Clínica implicados y la Agencia Española del Medicamento. Se sobreentiende que esta información no podrá ser revelada a otras personas, sin previa autorización por escrito del Promotor, excepto en la medida en que pueda ser necesario para obtener el consentimiento informado de las personas a las que se les pueda administrar el fármaco.

INDICE

página

1.- Indice ... 2
2.- Resumen del protocolo ... 3
3.- Identificación del Ensayo, Centros e Investigadores ... 5
4.- Justificación del estudio ... 6
5.- Bibliografía ... 9
6.- Objetivos ... 11
7.- Fuente de información y ámbito ... 11
8.- Tipo de estudio ... 11
8.1.-Proceso de aleatorización ... 11
8.2.-Criterios de inclusión ... 12
8.3.- Criterios de exclusión ... 12
8.4.- Criterios de retirada del Ensayo ... 12
8.5.-Tamaño de la muestra ... 13
8.6.- Duración del estudio ... 13
9.- Variables e instrumentos de medida ... 13
10.- Análisis estadístico ... 14
11.- Aspectos éticos ... 14
11.1.- Evaluación beneficio-riesgo para los participantes ... 14
11.2.- Hoja de información y consentimiento informado ... 14
11.3.- Confidencialidad de los datos ... 15
11.4.- Interferencia con los hábitos de prescripción ... 15
11.5.- Monitorización del ensayo ... 15
11.6.- Comités Éticos ... 16
11.7.-Condiciones para modificar el protocolo ... 16
11.8.-Condiciones para terminar el estudio ... 16
11.9.-Coservación de la documentación ... 16
12.-Consideraciones practicas ... 18
12.1.- Actuaciones tras inclusión en el ensayo y durante el seguimiento ... 18
12.2.- Dispensación y contabilización de la medicación del estudio ... 20
12.3.- Evaluación de la cumplimentación ... 20
12.4.- Medicación y tratamiento concomitante ... 20
13.- Acontecimientos adversos y anomalias de laboratorio ... 21
14.- Informes seguimientos, final y comunicación de los resultados ... 24
15.- Garantía de calidad ... 24
16.- Firma ... 24
Compromiso investigador coordinador (anexo 1) ... 25
Conformidad del CAEC (anexo 2) ... 26
Hoja información paciente y consentimiento informado (anexo 3) ... 27
Hoja notificación acontecimientos adversos (anexo 4) ... 29
Relación AA con medicación de estudio ... 31
Tablas gradación reacciones adversas ... 32
Declaración de Helsinki (anexo 5) ... 45
Normas de Buena Práctica Clínica (anexo 6) ... 49
Póliza de seguro (anexo 7) ... 50
Cuaderno recogida de datos (anexo 8) ... 53
Estudio económico (anexo 9) ... 57
Ficha técnica de Interferón-α 2a pegilado y ribavirina (anexo 10) ... 58

2.- RESUMEN DEL PROTOCOLO

**TITULO**	Eficacia de dosis bajas de interferón pegilado alfa-2a junto con ribavirina en el tratamiento de la hepatopatía crónica por virus C (genotipos 2 o 3) en pacientes coinfectados por el VIH
**PROMOTOR E INVESTIGADOR COORDINADOR**	Dr. Luis Fernando López Cortés.<br>Servicio de Enfermedades Infecciosas.<br>Avda. Manuel Siurot, s/n. 41013. Sevilla.<br>Tfno.: 955013096. Fax: 954234823.<br>e-mail: lflopez@telefonica.net<br>Fase IV
**INDICACION**	Pacientes con hepatopatía crónica por virus C (genotipos 2 o 3) coinfectados por el VIH.
**OBJETIVOS**	**Primario:**<br>▪ Evaluar la eficacia de dosis inferiores de interferón pegilado alfa-2a (135 µg/semana) junto con ribavirina (400 mg/12 h) y una duración inferior de tratamiento (20 semanas tras negativización de la viremia VHC) que las actualmente recomendadas en pacientes con hepatopatía crónica por virus C genotipos 2 o 3 coinfectados por el VIH en condiciones de uso habituales.<br><br>**Secundarios:**<br>▪ Evaluar la eficacia, medida como: respuesta viral precoz (4ª semana), respuesta viral temprana (12ª semana de tratamiento y al finalizar el tratamiento, de este régimen posológico.<br>▪ Evaluar la incidencia y severidad de los efectos adversos de este régimen posológico.<br>▪ Analizar la relación entre las concentraciones plasmáticas de IFN-α y ribavirina con la respuesta viral.<br>▪ Analizar la relación entre las concentraciones plasmáticas de IFN-α y ribavirina con la incidencia y gravedad de efectos adversos.
**DISEÑO**	Ensayo clínico piloto, abierto, postautorización.
**NUMERO DE PACIENTES**	71 pacientes, incluyendo unas pérdidas estimadas del 10%.
**CRITERIOS DE SELECCION**	**CRITERIOS DE INCLUSIÓN**<br>▪ Pacientes ≥ 18 años con infección por el VIH.<br>▪ Pacientes con hepatitis crónica o cirrosis por virus C (genotipos 2 o 3) que vayan a iniciar tratamiento con interferón-α pegilado 2a (Pegasys®) y ribavirina según criterio de su medico responsable.<br>▪ Test de embarazo en orina negativo (para mujeres en edad fértil y que puedan quedar embarazadas). Además, estas pacientes deben usar un método contraceptivo efectivo (incluye abstinencia sexual) durante el tratamiento. Ello no es necesario en mujeres esterilizadas quirúrgicamente o en estado de post-menopausia.<br>▪ Consentimiento informado del paciente.<br><br>**CRITERIOS DE EXCLUSIÓN**<br>▪ Tratamiento previo con interferón.<br>▪ Pacientes embarazadas o que no utilicen medios de contracepción seguros.<br>▪ Infección concomitante por el virus B de la hepatitis (HBsAg o DNA-VHB +).<br>▪ Aclaramiento de creatinina < 50 mL/min.<br>▪ Cirrosis hepática descompensada en el momento de la inclusión (ascitis, varices sangrantes, peritonitis, encefalopatía o hepatocarcinoma).<br>▪ Transplante de órgano.<br>▪ Alcoholismo o consumo activo de drogas por vía iv.<br>▪ Tratamiento concomitante con inmunomodulares, didanosina (contraindicación absoluta) o zidovudina (contraindicación relativa).<br>▪ Existencia de un proceso psiquiátrico grave, en especial depresión grave e ideas o intento de suicidio.<br>▪ Enfermedades autoinmunes concomitantes<br>▪ Pacientes no capacitados o incapaces de otorgar el consentimiento legal.<br>▪ Para el análisis de los resultados se excluirán aquellos pacientes que no tengan al menos una visita clínica y/o analitica de seguimiento
**DURACION DEL ENSAYO**	Enero 2008 a abril 2010<br>▪ Periodo de inclusión: 52 semanas<br>▪ Periodo de tratamiento máximo: 44 semanas<br>▪ Periodo de seguimiento: 24 semanas
**DOSIS / VIA / REGIMEN DE LOS**	Interferón pegilado alfa-2a subcutáneo (135 µg/semana) + ribavirina oral 400 mg/12 horas) con una duración de 20 semanas tras la negativización de la viremia plasmática VHC medida por PCR cualitativa.

**FARMACOS EN INVESTIGACION**	
**EVALUACIONES DE:**	
**1.- SEGURIDAD**	La incidencia y severidad de efectos adversos se evaluará mediante entrevista clínica, exploración y parámetros de laboratorio. Su intensidad se medirá mediante una escala de efectos adversos (Division of AIDS. Table for grading the severity of adult and pediatric adverse events. Diciembre, 2004). Asimismo, se recogerá el uso de factores hematoestimulantes (eritropoyetina y GCS-F).
**2.- EFICACIA**	La variable de eficacia para el objetivo principal se evaluará como como porcentaje de pacientes con respuesta viral sostenida (riremia VHC indetectable en plasma mediante PCR cualitativa) 24 semanas tras finalizar el tratamiento. Asímismo se evaluaran la respuesta viral rápida (4 semanas de tratamiento), respuesta viral temprana (12 semanas de tratamiento) y la respuesta al finalizar el tratamiento.
**ANÁLISIS ESTADÍSTICO**	Estadística descriptiva para datos de farmacocinética, de eficacia (analizada por intención de tratar y en tratamiento), tolerancia y seguridad. La población que se incluirá en el análisis de eficacia y de efectos adversos estará constituida por todos aquellos pacientes que reciban al menos una dosis de tratamiento y tengan al menos una visita clínica y/o analitica de seguimiento. La relación entre concentraciones plasmáticas de ambos fármacos y eficacia, así como con otras variables, se realizará mediante el test de Chi cuadrado en caso de variables cualitativas y mediante coeficientes de correlación de Spearman en el caso de variables cuantitativas. Se realizará un análisis de regresión logística múltiple integrando las distintas variables predictoras de respuesta.
**FUENTE DE FINANCIACIÓN**	Dado que el promotor no dispone de medios económicos para la realización del ensayo clínico, éste como investigador coordinador y el resto los investigadores solicitarán financiación para la realización del ensayo en convocatoria/s pública/s de agencias oficiales. En caso de la subvención concedida no cubra la totalidad de los gastos, se solicitarán otras ayudas económicas.

**Evaluación**	**Semanas**						
	**0**	**1**	**4**	**12**	**24***	**36***	**24 semanas tras fin tratº***
Consentimiento informado	x						
Test de embarazo	x		x	x	x	x	
Medicación concomitante	x	x	x	x	x	x	
Hematología	x	x	x	x	x	x	x
Bioquímica	x	x	x	x	x	x	x
TSH ± hormonas tiroideas	x		x	x	x	x	x
Genotipo VHC	x						
RNA-VHC UI/ml	x	x	x	x	x	x	x
RNA-VHC cualitativa		x	x	x	x	x	x
HIV-1-RNA	x		x	x	x	x	x
Recuento de CD4+	x		x	x	x	x	x
Cumplimentación tratamiento		x	x	x	x	x	x
Concentraciones plasmáticas de Interferón y Ribavirina		x	x	x	x	x	

***:** en función duración de tratamiento con interferón pegilado-α 2a y ribavirina

3.- IDENTIFICACIÓN DEL ENSAYO

Código de protocolo: SAEI_IFN_1

Nº EudraCT: **2007-000814-35**

Título: Eficacia de dosis bajas de interferón pegilado alfa-2a junto con ribavirina en el tratamiento de la hepatopatía crónica por virus C (genotipos 2 o 3) en pacientes coinfectados por el VIH

Tipo de ensayo clínico: Ensayo clínico, abierto, fase IV, postautorización.

Descripción de los productos en estudio

Interferón pegilado alfa-2a y ribavirina (especialidades farmacéuticas autorizadas y comercializadas en España para esta indicación).

Datos relativos al promotor e investigador coordinador

Dr. Luis Fernando López Cortés.
Servicio de Enfermedades Infecciosas.
Hospitales Universitarios Virgen del Rocío.
Avda. Manuel Siurot, s/n. 41013. Sevilla.
Tfno.: 955013094. Fax: 954234823. e-mail: lflopez@telefonica.net

Identificación del monitor

Alicia Gutierrez Valencia. Licenciada en Farmacia. Becaria de la Fundación Reina Mercedes. Hospitales Universitarios Virgen del Rocío

Centros donde se prevé realizar el Ensayo e investigadores principales en cada centro:

H.U. Reina Sofía	Córdoba	Dr. Antonio Rivero Román
H.U. Virgen de Valme	Sevilla	Dr. Angel Garcia Garcia
H. U. Virgen del Rocío	Sevilla	Dr. Luis F. López Cortés
H.U. Virgen de la Victoria	Malaga	Dra. Mercedes González Serrano

Duración prevista del Ensayo:

118 semanas.

4.- JUSTIFICACION DEL ESTUDIO

La hepatitis crónica por virus de la hepatitis C (VHC) supone un importante problema de salud pública a escala mundial, que resulta especialmente relevante en pacientes infectados por el VIH debido a que ambos virus comparten el mismo mecanismo de transmisión (1), estimandose que más del 50% de los sujetos infectados por el VIH se encuentran coinfectados por el VHC en España (2).

El tratamiento antirretroviral actual ha dado lugar a un espectacular aumento de la supervivencia de los pacientes infectados por el VIH y, a la vez, ha permitido que comorbilidades como la hepatitis crónica por VHC progrese a situación terminal en muchos pacientes pasando a ser una de las causas mas frecuentes de hospitalización y muerte en estos pacientes en áreas de alta incidencia de coinfección VIH/VHC. Ello origina que el tratamiento de la hepatitis crónica por VHC sea un elemento de gran importancia para mejorar la supervivencia a largo plazo de los pacientes coinfectados por VIH/VHC.

El tratamiento estándar actual para la hepatitis crónica por virus C (VHC) se basa en la combinación de interferón pegilado-alfa (pegIFN-α) 2a (180 µg/semana) ó 2b (1,5 µg/kg/semana) semanal junto con ribavirina (Rbv) dosificada en función del genotipo (G) del VHC (1000 - 1200 mg/día para los G 1 o 4 y 800 mg/día para los G 2 o 3) durante 48 semanas para pacientes con G 1 o 4 del VHC, independientemente de que estén coinfectados por el VIH (1-4). Aunque los pacientes coinfectados tienen un aclaramiento de la viremia VHC más lento (5), se ha observado que la prolongación a 72 semanas no aumenta las tasas de éxito en estos pacientes (6).

Por otro lado, los G 2 y 3 del VHC tienen una sensibilidad mucho mayor al tratamiento y unas tasas de RVR, RPT y RVS mucho más elevadas que otros G hasta el punto que se recomiendan unas dosis menores de Rbv y una duración de tratamiento de sólo 24 semanas en pacientes no coinfectados por el VIH (3), habiendose observado que incluso sólo 14 semanas de tratamiento pueden ser suficientes en los pacientes que negativizan la viremia en la 4ª semana de tratamiento (7). Aunque en pacientes coinfectados por el VIH se recomienda prolongar el tratamiento hasta 48 semanas, esta recomendación se basa en el diseño de los escasos ensayos clínicos que se han realizado en esta población, en los que sólo se ha probado esta duración de tratamiento, más que en estudio comparativos que no existen (9,10).

Además de ser un tratamiento largo, con frecuentes efectos adversos que obligan a su retirada o a disminuir las dosis, las tasas de respuesta viral sostenida (RVS: no detección de ARN-VHC en plasma 6 meses después de finalizado el tratamiento) en aquellos pacientes que completan el tratamiento estan lejos de ser óptimas ya sea por no respuesta o por recidivas, sobre todo en casos de pacientes con G 1 o 4 (11-13).

El genotipo y la respuesta viral durante las 12 primeras semanas de tratamiento (RVT) son los mejores factores predictivos de RVS hasta el punto que, si la viremia no disminuye ≥ 2 $\log_{10}$ en la semana 12 de

tratamiento (RVT: respuesta viral temprana), éste se suspende dadas las escasas posibilidades de conseguir una RVS (3,4). Asímismo, se ha observado que la negativización de la viremia en la semana 4 (RVR: respuesta viral rápida) tiene un valor predictivo positivo muy elevado para alcanzar una RVS, independientemente del G y de la coinfección por el VIH, incluso con solo 24 semanas de tratamiento en pacientes con G 1 y cargas virales bajas (14-19).

La dosis actualmente recomendadas de pegIFN-α 2a es de 180 µg/semana, independientemente del G del VHC (22). Con esta dosis las RVS fueron algo más frecuentes que con 135 µg/semana en pacientes con G 1, aunque las respuestas virales al final del tratamiento (RFT) parecen ser similares con 135 o 180 µg/semana (11,21-23). En cambio, para los pacientes con G 2 o 3, las tasas de RVS fueron similares con dosis de 90, 135 o 180 µg/semana (21,22). Asimismo, con pegIFN-α 2b, las tasas de RVS fueron iguales (73%) en pacientes con G 2 o 3 tratados durante 24 semanas independientemente de la dosis de pegIFN (0,75 µg/kg/semana y 1,5 µg/kg/semana) a pesar de tratarse de pacientes con fibrosis hepática severa o cirrosis (24).

Respecto a Rbv, sola no tiene una actividad sustancial sobre el VHC pero sí tiene un papel sinérgico junto con IFN y actualmente es un componente fundamental del tratamiento aunque su mecanismo de acción exacto permanece desconocido (3,11-13). Dado que el VHC no se ha conseguido cultivar eficientemente, no se conoce su susceptibilidad a Rbv ni la/s dosis o concentraciones óptimas para los distintos genotipos. Además, no hay datos en la bibliografía sobre la correlación entre sus concentraciones plasmáticas y los niveles de Rbv intracelular trifosforilada en células nucleadas (el metabolito activo y el verdadero compartimento del efecto, respectivamente) o de las concentraciones intracelulares de Rbv intracelular trifosforilada necesarias para ejercer su efecto sobre los distintos G del VHC. De hecho, su dosificación se ha basado en la dosis utilizada para tratar otros virus como el virus sincitial respiratorio. Dosis elevadas de Rbv (1000–1200 mg/día) se han asociado con mayores probabilidades de obtener una RVS sólo en el caso de G 1 o 4 pero la incidencia y gravedad de sus efectos adversos se asocian con las dosis y las concentraciones plasmáticas más altas (25-27). Además, algunos estudios sugieren que dosis más bajas podrían ser igualmente eficaces en los G 2 y 3 (28-30).

Dado que, con cualquiera de las dosis de pegIFN-α y Rbv, existe una gran variabilidad interindividual en las concentraciones de ambos fármacos, independientemente del sexo, peso u otras variables (20,31), recientemenet hemos evaluado el papel de las concentraciones de pegIFN-α 2a y Rbv en la RVT en pacientes coinfectados con VHC/VIH, constatando que las únicas variables relacionadas con la RVT fueron el G y las concentraciones plasmáticas de pegIFN-α 2a. Cuando estratificamos por G del VHC, la probabilidad de obtener una RVT en los G 1 y 4 fue más de 4 veces superior en aquellos pacientes con

concentraciones de pegIFN-α 2a por encima del percentil 25. En cambio, para los G 3, la respuesta fue independiente de las concentraciones (32).

Dado que la eficacia de IFN-α para inhibir la replicación viral in vitro es mucho mayor para los G 2 y 3 que para otros genotipos, las tasas de RVS similares con dosis de 90, 135 o 180 µg/semana de pegIFN-α 2a o de 0,75 o 1,5 µg/kg/semana de pegIFN-α 2b y la falta de relación entre las concentraciones de pegIFN-α 2a y la RVT sugieren que la dosis de 180 µg/semana de pegIFN-α 2a da lugar a concentraciones que se encuentran en la parte más alta de la curva de concentraciones-respuesta.

Ya que tanto PegIFN-α como ribavirina tienen importantes efectos secundarios, un objetivo debe ser encontrar la dosis minima más eficaz y la menor duración posible del tratamiento. En este sentido, pensamos que en pacientes con G 2 o 3 la eficacia de regímenes basados en dosis inferiores de pegIFN-α 2a que las actualmente recomendadas y una duración de 20 semanas tras la negativización de la viremia VHC podrían ser igual de eficaces pero con una incidencia y gravedad de efectos adversos menores y, por tanto, con un porcentaje mayor de finalizaciones de tratamiento y de éxitos terapéuticos.

Un ensayo clínico definitivo con 2 brazos requiriría la participación de múltiples investigadores y centros para reclutar un mínimo de 260 pacientes por brazo sin tener en cuenta las posibles pérdidas. Este número de pacientes es dificil de reclutar sin datos preliminares que apoyen la dosificación de dosis menores de interferón-α pegilado y ribavirina en los pacientes con hepatitis crónica por virus C coinfectados por el VIH en condiciones de uso reales. Por ello, hemos diseñado un ensayo clínico piloto para evaluar la eficacia de éste régimen.

5.- BIBLIOGRAFIA

1. Soriano V, Sulkowski M, Bergin C, Hatzakis A, Cacoub P, Katlama C, et al. Care of patients with chronic hepatitis C and HIV co-infection: recommendations from the HIV-HCV International Panel. AIDS 2002;16:813-828.
2. Gonzalez-Garcia JJ, Mahillo B, Hernandez S, Pacheco R, Diz S, Garcia P, et al. Estudio multicéntrico sobre prevalencia de las coinfecciones por virus de hepatitis, indicación de tratamiento de hepatitis crónica C y necesidad de trasplante hepático en pacientes infectados por el VIH en España. Estudio GESIDA 29/02-FIPSE 12185/01. Enferm Infecc Microbiol Clin 2005;23:340-348.
3. Strader DB, Wright T, Thomas DL, Seeff LB. Diagnosis, management, and treatment of hepatitis. AASLD practice guideline. Hepatology 2004;39:1147-1171.
4. Varios Autores. Proceedings of the 1st European Consensus Conference on the Treatment of Chronic Hepatitis B and C in HIV Co-infected Patients. Editores: Benhamou Y, Salmon D. March 2005. Paris, France J Hepatol 2006;44 (suppl 1): S1-S152.
5. Torriani F, Ribeiro R, Gilbert T, Schrenk U, Clauson M, Pacheco D, et al. HCV and HIV dynamics during HCV treatment in HCV/HIV coinfection. J Infect Dis 2003;188:1498-1507.
6. Uriel A, Moorehead L, Carriero D, Sulkowski M, Dieterich D, and Hepatitis Resource Network Clin Trials Group. A Multicenter, Randomized Trial of 48 vs 72 Weeks of Peg-Interferon Alfa -2b + Ribavirin in HIV/HCV-co-infected Subjects: Longer Therapy Does Not Correlate with Improved Sustained Virological Response. 13th Conference on Retroviruses an Opportunistic Infections. Februari 5 - 8, 2006. Denver. USA. Abstrac # 854.
7. Neumann AU, Lam NP, Dahari H, Davidian M, Wiley TE, Mika BP, et al. Differences in viral dynamics between genotypes 1 and 2 of hepatitis C virus. J Infect Dis. 2000;182:28-35.
8. Dalgard O, Bjoro K, Hellum KB, Myrvang B, Ritland S, Skaug K, Raknerud N, Bell H. Treatment with pegylated interferon and ribavarin in HCV infection with genotype 2 or 3 for 14 weeks: a pilot study. Hepatology. 2004;40:1260-1265.
9. Torriani FJ, Rodriguez-Torres M, Rockstroh JK, Lissen E, Gonzalez-Garcia J, Lazzarin A, et al. APRICOT Study Group. Peginterferon Alfa-2a plus ribavirin for chronic hepatitis C virus infection in HIV-infected patients. N Engl J Med 2004;351:438-450.
10. Chung RT, Andersen J, Volberding P, Robbins GK, Liu T, Sherman KE, et al. AIDS Clinical Trials Group A5071 Study Team. Peginterferon Alfa-2a plus ribavirin versus interferon alfa-2a plus ribavirin for chronic hepatitis C in HIV-coinfected persons. N Engl J Med 2004;351:451-459.
11. Zeuzem S, Feinman SV, Rasenack J, Heathcote EJ, Lai MY, Gane E, et al. Peginterferon alfa-2a in patients with chronic hepatitis C. N Engl J Med 2000;343:1666-1672.
12. Heathcote EJ, Shiffman ML, Cooksley WG, Dusheiko GM, Lee SS, Balart L,et al. Peginterferon alfa-2a in patients with chronic hepatitis C and cirrhosis. N Engl J Med 2000;343:1673-80.
13. Fried MW, Shiffman ML, Reddy KR, Smith C, Marinos G, Goncales FL Jr, et al. Peginterferon alfa-2a plus ribavirin for chronic hepatitis C virus infection. N Engl J Med 2002;347:975-982.
14. Carlsson T, Reichard O, Norkrans G, Blackberg J, Sangfelt P, Wallmark E, et al. Hepatitis C virus RNA kinetics during the initial 12 weeks treatment with pegylated interferon-alpha 2a and ribavirin according to virological response. J Viral Hepat. 2005;12:473-480.
15. Jensen DM, Morgan TR, Marcellin P, Pockros PJ, Reddy KR, Hadziyannis SJ, et al. Early identification of HCV genotype 1 patients responding to 24 weeks peginterferon alpha-2a (40 kd)/ribavirin therapy. Hepatology. 2006;43:954-960.
16. Dieterich D, Duff F, Sulkowski M, Torriani F, Lissen E, Brau N, et al. Sustained virologic response (SVR) in HIV-HCV co-infected patients with HCV genotype 1 infection who have a rapid virological response (RVR) at week 4 of treatment with peginterferon alfa-2a (40KD) plus ribavirin. 13th Conference on Retroviruses and Opportunistic Infections. February 5 - 8, 2006. Denver. USA. Abstract #856.

17. Zeuzem S, Buti M, Ferenci P, Sperl J, Horsmans Y, Cianciara J, et al. Efficacy of 24 weeks treatment with peginterferon alfa-2b plus ribavirin in patients with chronic hepatitis C infected with genotype 1 and low pretreatment viremia. J Hepatol. 2006;44:97-103.
18. Von Wagner M, Huber M, Berg T, Hinrichsen H, Rasenack J, Heintges T et al. Peginterferon-α-2a (40 KD) and ribavirin for 16 or 24 weeks in patients with genotype 2 or 3 chronic hepatitis C. Gastroenterology 2005;129:522-527.
19. Shiffman ML et al. PegInterferon alfa-2a plus ribavirin for 16 or 24 weeks in patients with HCV genotype 2 or 3. Final results of the ACCELERATE Trial. EASL 2006. J Hepatol 2006; 44:734A
20. Pegasys®. Ficha técnica del producto. Roche Farma S.A. Agencia Europea del medicamento. Disponible en: http://www.emea.eu.int/newdomain.htm.
21. Reddy KR, Wright TL, Pockros PJ, Shiffman M, Everson G, Reindollar R, et al. Efficacy and safety of pegylated (40-kd) interferon alpha-2a compared with interferon alpha-2a in noncirrhotic patients with chronic hepatitis C. Hepatology 2001;33:433-438.
22. Pockros PJ, Carithers R, Desmond P, Dhumeaux D, Fried MW, Marcellin P, et al. Efficacy and safety of two-dose regimens of peginterferon alpha-2a compared with interferon alpha-2a in chronic hepatitis C: a multicenter, randomized controlled trial. Am J Gastroenterol. 2004;99:1298-1305.
23. Hadziyannis SJ, Sette H, Morgan TR, Balan V, Diago M, Marcellin P, et al. Peginterferon-alpha-2a (40 kilodaltons) and ribavirin combination therapy in chronic hepatitis C: a randomized study of treatment duration and ribavirin dose. Ann Intern Med 2004;140:346-355.
24. Abergel A, Hezode C, Leroy V, Barange K, Bronowick JP, Tran A. Peginterferon alpha-2b plus ribavirin for treatment of chronic hepatitis C with severe fibrosis: a multicentre randomized controlled trial comparing two doses of peginterferon alpha-2b. J Viral Hepat. 2006;13:811-820.
25. Lindahl K, Schvarcz R, Bruchfeld A, Stahle L. Evidence that plasma concentration rather than dose per kilogram body weight predicts ribavirin-induced anaemia. J Viral Hepat 2004;11:84-87.
26. Rendon AL, Nunez M, Romero M, Barreiro P, Martin-Carbonero L, Garcia-Samaniego J, et al. Early monitoring of ribavirin plasma concentrations may predict anemia and early virologic response in HIV/hepatitis C virus-coinfected patients. J Acquir Immune Defic Syndr 20051;39:401-405.
27. Bonkovsky HL, Stefancyk D, McNeal K, Banner BF, Liu Q, Zucker GM, et al. Comparative effects of different doses of ribavirin plus interferon-alpha2b for therapy of chronic hepatitis C: results of a controlled, randomized trial. Dig Dis Sci. 2001;46:2051-2059.
28. Hrachovec J, Patel P. Weight-based ribavirin with pegylated interferon alfa 2b: benefits, risks, and the origin of the evidence. Gastroenterology 2003;124:2003-2004.
29. Ferenci E, Brunner H, Laferl H, Bergholz U, Rosenbeigel M, Stauber R, et al. Further reduction of ribavirin dose in HCV genotype 2/3 patients receiving peginterferon alpha-2a (40kd) plus ribavirin: interim results of a randomised controlled trial. J Hepatol 2006;44:S37.
30. Larrat S, Stanke-Labesque F, Plages A, Zarski JP, Bessard G, Souvignet C. Ribavirin quantification in combination treatment of chronic hepatitis C. Antimicrobial Agents Chemotherapy 2003;47:124-1299.
31. Jen JF, Glue P, Gupta S, Zambas D, Hajian G. Population pharmacokinetic and pharmacodynamic analysis of ribavirin in patients with chronic hepatitis C. Ther Drug Monit 2000;22:5555-5565.
32. Lopez-Cortes LF, Valera B, Gutierrez A, Ruiz-Valderas R, Jimenez L, Arizcorreta A, et al. Role of pegylated interferon-α 2a and ribavirin plasma concentrations in achieving an HCV early virological response in HIV-coinfected patients. Pendiente de publicación.

6.- OBJETIVOS

Primario:

- Evaluar la eficacia (*) de dosis inferiores de interferón pegilado alfa-2a (135 µg/semana) junto con ribavirina (400 mg/12 h) y una duración inferior de tratamiento (20 semanas tras negativización de la viremia VHC#) que las actualmente recomendadas en pacientes con hepatopatía crónica por virus C genotipos 2 o 3 coinfectados por el VIH en condiciones de uso habituales.

(*): medida como respuesta viral sostenida (viremia indetectable 24 semanas tras finalizar el tratamiento).

(#):respuesta viral: negativización de viremia en semana 4, 12 o 16 semanas de tratamiento. En el último caso se refiere a pacientes con disminución $\geq 2 \log_{10}$ respecto a la basal tras 12 semanas de tratamiento.

Secundarios:

- Evaluar la eficacia, medida como: respuesta viral precoz (4ª semana), respuesta viral temprana (12ª semana de tratamiento y al finalizar el tratamiento, de este régimen posológico.
- Evaluar la incidencia y severidad de los efectos adversos de este régimen posológico.
- Analizar la relación entre las concentraciones plasmáticas de IFN-α y ribavirina con la respuesta viral.
- Analizar la relación entre las concentraciones plasmáticas de IFN-α y ribavirina con la incidencia y gravedad de efectos adversos.
- Analizar la relación entre las concentraciones plasmáticas e intracelulares de ribavirina.

7.- FUENTE DE INFORMACIÓN Y ÁMBITO

La fuente de información es primaria, es decir directamente de los pacientes, así como de las historias clínicas y pruebas de laboratorio. En todo caso, se solicitará el consentimiento informado del paciente y se garantizará la protección de sus datos personales El ámbito es hospitalario y autonómico (Hospitales del Servicio Andaluz de Salud por determinar). El ensayo clínico cuenta con la aprobación de la Sociedad Andaluza de Enfermedades Infecciosas.

8.- TIPO DE ESTUDIO

Ensayo clínico, abierto, fase IV, postautorización.

Interferón pegilado alfa-2a (L03AB11) y ribavirina (J05A B04) son especialidades farmacéuticas autorizadas y comercializadas en España para esta indicación.

8.1.- PROCESO DE ALEATORIZACION

No procede.

8.2.- CRITERIOS DE INCLUSIÓN

- Pacientes ≥ 18 años con infección por el VIH.
- Pacientes con hepatitis crónica o cirrosis compensada por virus C (genotipos 2 o 3) que vayan a iniciar tratamiento con interferón-α pegilado 2a (Pegasys®) y ribavirina según criterio de su medico responsable.
- Test de embarazo en orina negativo (para mujeres en edad fértil y que puedan quedar embarazadas). Además, estas pacientes deben usar un método contraceptivo efectivo (incluye abstinencia sexual) durante el tratamiento. Ello no es necesario en mujeres esterilizadas quirúrgicamente o en estado de post-menopausia.
- Consentimiento informado del paciente.
- Aunque los datos del seguimiento prospectivo de, aproximadamente, 300 embarazos con exposición paterna a ribavirina no han demostrado un incremento del riesgo de malformaciones comparado con la población en general, ni ningún modelo específico de malformación, los pacientes masculinos y sus parejas femeninas en edad fértil deben usar un método anticonceptivo eficaz durante el tratamiento con ribabvirina y durante siete meses después del tratamiento.

8.3.- CRITERIOS DE EXCLUSIÓN

- Tratamiento previo con interferón.
- Pacientes embarazadas o que no utilicen medios de contracepción seguros.
- Infección concomitante por el virus B de la hepatitis (HBsAg o DNA-VHB +).
- Aclaramiento de creatinina < 50 mL/min.
- Cirrosis hepática descompensada en el momento de la inclusión (ascitis, varices sangrantes, peritonitis, encefalopatía o hepatocarcinoma).
- Transplante de órgano.
- Alcoholismo o consumo activo de droga, excluyendo tabaco.
- Tratamiento concomitante con inmunomodulares.
- Existencia de un proceso psiquiátrico grave, en especial depresión grave e ideas o intento de suicidio.
- Enfermedades autoinmunes concomitantes.
- Pacientes no capacitados o incapaces de otorgar el consentimiento legal.
- Para el análisis de los resultados se excluirán aquellos pacientes que no tengan al menos una visita clínica y/o analitica de seguimiento.

8.4.- CRITERIOS DE RETIRADA DEL ESTUDIO

- Imposibilidad para seguir las visitas programadas en el plan de trabajo
- Constatación de no cumplimentación del tratamiento prescrito.
- Aparición de efectos adversos graves, que hagan necesaria la retirada de los fármacos en estudio.
- Decisión del investigador ó del paciente.

8.5.- TAMAÑO DE LA MUESTRA

Dado que un ensayo de no inferioridad sería difícilmente viable por dificultades para alcanzar el tamaño muestral adecuado, este estudio se ha diseñado para estimar una proporción, con una precisión prefijada. Para ello, la proporción elegida ha sido el 62%, que fue la tasa de respuesta viral sostenida obtenida en el ensayo Apricot(*) en pacientes con genotipo 2-3, y la precisión del 10. Así, podríamos obtener una frecuencia que teóricamente cayera dentro de un intervalo de no inferioridad razonable, si el estudio se hubiera diseñado de ese modo. Con arreglo a estos parámetros, será necesario incluir 63 pacientes en el estudio para conseguir la precisión indicada con un intervalo de confianza asintótico normal bilateral del 95%, por lo que asumiendo unas pérdidas del 10%, el número total de pacientes sería de 71.

.

(*): Torriani FJ, et al. APRICOT Study Group. Peginterferon Alfa-2a plus ribavirin for chronic hepatitis C virus infection in HIV-infected patients. N Engl J Med 2004;351:438-450.

8.6.- DURACIÓN DEL ESTUDIO

- Enero 2008 a abril 2010
  - Periodo de inclusión: 52 semanas
  - Periodo de tratamiento máximo: 44 semanas
  - Periodo de seguimiento: 24 semanas

.

9.- VARIABLES E INSTRUMENTOS DE MEDIDA

1. La variable de eficacia para el objetivo principal se evaluará como como porcentaje de pacientes con respuesta viral sostenida (viremia VHC indetectable en plasma) 24 semanas tras finalizar el tratamiento. Asímismo se evaluaran la respuesta viral rápida (4 semanas de tratamiento), respuesta viral temprana (12 semanas de tratamiento) y la respuesta al finalizar el tratamiento.
2. La incidencia y severidad de efectos adversos se evaluará mediante entrevista clínica, exploración y parámetros de laboratorio. Su intensidad se medirá mediante una escala de efectos adversos (Division of AIDS. Table for grading the severity of adult and pediatric adverse events. Diciembre, 2004). Asimismo, se recogerá el uso de factores hematoestimulantes (eritropoyetina y GCS-F).
3. Las determinaciones de las concentraciones plasmáticas de interferón-α y de ribavirina se llevarán a cabo mediante cromatografía liquida con detector de espectrometría de masas y ELISA basado en anticuerpos monoclonales (ELISA Hu-INF-α® -PBL Biomedical Laboratories, New Jersey. USA), respectivamente.
4. Otras variables: evaluaciones clínicas y analíticas habituales en el seguimiento de estos pacientes, incluyendo hemograma, función hepática, subpoblaciones linfocitarias, carga viral plasmática VIH.

10.- ANÁLISIS ESTADÍSTICO

Estadística descriptiva para datos de farmacocinética, de eficacia (analizada por intención de tratar y en tratamiento), tolerancia y seguridad. La población que se incluirá en el análisis de eficacia y de efectos adversos estará constituida por todos aquellos pacientes que reciban al menos una dosis de tratamiento y tengan al menos una visita clínica y/o analitica de seguimiento. La relación entre concentraciones plasmáticas de ambos fármacos y eficacia, así como con otras variables, se realizará mediante el test de Chi cuadrado en caso de variables cualitativas y mediante coeficientes de correlación de Spearman en el caso de variables cuantitativas. Se realizará un análisis de regresión logística múltiple integrando las distintas variables predictoras de respuesta.

11.- ASPECTOS ÉTICOS

11.1.- Evaluación beneficio-riesgo para los sujetos de la investigación:

En base a la evidencia actualmente disponible, el riesgo de efectos adversos para los sujetos incluidos en el ensayo clínico es inferior al de realizar un tratamiento con interferón pegilado-α 2a y ribavirina a las dosis habitualmente utilizadas en la práctica clínica.

Los resultados de este estudio pueden ayudar a conocer si dosis inferiores de los fármacos habitualmente empleados en el tratamiento de la hepatopatía crónica por virus C son igualmente eficaces. En ese caso es de esperar una menor incidencia y gravedad de efectos adversos, menores porcentajes de reducciones o retirada de tratamientos y, por tanto, un mayor porcentaje de finalizaciones de tratamiento y de éxitos terapéuticos con un coste menor. Los resultados pueden ser aplicables tanto a pacientes coinfectados por el VIH como a aquellos infectados solamente por el VHC.

El estudio se realizará de acuerdo a los Principios de Buena Práctica Clínica (Ministerio de Sanidad y Consumo. Real Decreto 223/2004, de 6 de febrero) y a la Declaración de Helsinki (anexo 5).

11.2.- Hoja de información y consentimiento informado: Se adjunta como anexo 3.

El investigador, o una persona designada por él, es responsable de obtener el consentimiento informado por escrito de cada paciente que participe en este estudio después de haber proporcionado una explicación adecuada de los objetivos, métodos, beneficios anticipados y riesgos potenciales. En el caso en el que el paciente sea incapaz de leer, un testigo imparcial deberá estar presente durante todo el proceso de consentimiento informado. Después de que el paciente haya otorgado verbalmente su consentimiento para participar en el ensayo, el testigo firmará el formulario para dar fe de que la información contenida en el formulario de consentimiento se ha explicado y entendido exactamente. El investigador o la persona designada también deben explicar que los pacientes son completamente libres de negarse a participar en el estudio o retirarse del mismo en cualquier momento, por cualquier motivo. Los CRD de este estudio contienen una sección para documentar el consentimiento informado del sujeto, que se debe completar adecuadamente. Si se obtiene información nueva sobre la seguridad que pueda dar lugar a cambios significativos en la evaluación del riesgo/beneficio, se deberá revisar y actualizar el formulario de consentimiento, si fuera necesario. La información nueva debe comunicarse a todos los sujetos (incluidos

los que ya se están tratando) y se les proporcionará una copia del formulario revisado y deberán otorgar su consentimiento para continuar en el estudio.

11.3.- Confidencialidad de los datos:

Los datos del estudio se transcribirán a formularios de recogida de datos en los que sólo figurarán las iniciales de las pacientes y un código asignado para cada paciente, que servirá de código de identificación para pacientes y muestras de sangre. Únicamente los médicos y enfermeros/as participantes en el estudio y el personal autorizado por los organismos oficiales, en caso necesario, podrán tener acceso a la historia clínica de los pacientes según la Ley 15/1999, de 13 de diciembre de Protección de datos personales.

Esta información confidencial será de la exclusiva propiedad del investigador principal, no podrá ser revelada a otros sin previo consentimiento escrito del investigador y no podrá ser utilizada excepto para la realización de este estudio.

La información que se vaya creando durante la realización de este estudio clínico es también considerada confidencial y será utilizada por los investigadores en relación con los objetivos del estudio y el desarrollo del medicamento del estudio.

11.4.- Interferencia con los hábitos de prescripción de los médicos:

No hay interferencia con los hábitos de prescripción del médico dado el diseño del estudio y que interferón pegilado-α 2a y ribavirina (fármaco genérico) son los fármacos admitidos y utilizados habitualmente en los hospitales participantes para el tratamiento de la hepatopatía crónica por virus C en estos pacientes.

11.5.- Monitorizacion del estudio

El monitor responsable contactará y visitará regularmente al investigador y se le permitirá, si así lo solicita, inspeccionar los diversos registros del ensayo (CRD y otros datos pertinentes), siempre que se mantenga la confidencialidad de los pacientes, de acuerdo con los requisitos locales.

El monitor será responsable de inspeccionar los CRD a intervalos regulares durante el estudio, para verificar el cumplimiento con el protocolo y que los datos incluidos son completos, consistentes y exactos. El monitor debe tener acceso a los informes de las pruebas de laboratorio y otros registros de los pacientes que sean necesarios para verificar la información incluida en el CRD. El investigador (o su representante) acepta colaborar con el monitor para asegurar que se resuelven todos los problemas detectados en el transcurso de estas visitas de monitorización.

11.6.- Comités Eticos /Comité de Investigación Clínica

El promotor e investigador coordinador del ensayo presentará al Comité Autonómico de Ensayos Clínicos de Andalucía este protocolo y todos los materiales relacionados que se proporcionen al sujeto (tales como hojas del información al sujeto o descrpciones del estudio utilizadas para obtener el consentimiento

informado), así como cualquier compensación proporcionada al paciente. Se debe obtener la aprobación del citado Comité y de la Agencia Española del Medicamento antes de iniciar el estudio y se debe documentar en una carta al investigador, en la que se especifique la fecha en la que éstos se reunieron y otorgó su aprobación.

El investigador también debe presentar al Comité cualquier modificación realizada en el protocolo después de recibir la aprobación, de acuerdo con los procedimientos y requisitos legales locales.

El promotor sobreentiende que este protocolo (y cualquier modificación), así como los procedimientos de consentimiento apropiados, serán revisados y aprobados por el Comité Autonómico de Ensayos Clínicos de Andalucía.

11.7.- Condiciones para modificar el protocolo

Las modificaciones del protocolo para estudios en curso que posiblemente pudiesen afectar de manera adversa a la seguridad de los pacientes participantes o alterar el ámbito de la investigación, la calidad científica del estudio, el diseño experimental, las dosis, la duración de la terapia, las variables de evaluación, el número de pacientes tratados o los criterios de selección de los pacientes, se deberán realizar únicamente después de la consulta entre el promotor y el investigador.

El promotor debe preparar las modificaciones del protocolo.

11.8.- Condiciones para terminar el estudio

El promotor se reserva el derecho a terminar el estudio en cualquier momento. Si esto fuera necesario, se decidirán los procedimientos de forma individual, después de la revisión y consulta por ambas partes. A la hora de terminar el estudio, el promotor y los investigadores asegurarán que se presta debida consideración a la protección de los intereses de los pacientes.

11.9.- Conservacion de la documentacion del estudio

Archivos del investigador / Conservación de los documentos

El Investigador debe mantener un registro adecuado y exacto para permitir que el estudio esté totalmente documentado y que se puedan verificar posteriormente los datos del estudio. Estos documentos se deben clasificar en dos categorías diferentes (1) el archivo del estudio del investigador y (2) la documentación clínica original del sujeto.

El archivo del estudio del investigador contendrá el protocolo/enmiendas, los cuadernos de recogida de datos y dudas, la aprobación oficial y del CEI/CIC con la correspondencia, el modelo de consentimiento informado, los registros de la medicación, los curriculum vitae del equipo, las hojas de autorización y otros documentos/correspondencia apropiados, etc.

La documentación clínica original del sujeto (normalmente definida antes del proyecto para registrar los parámetros principales de eficacia/seguridad independientes de los CRD) incluirían las historias del hospital/clínica del paciente, las notas del médico y la enfermera, los informes originales de laboratorio, ECG, EEG, radiografías, informes patológicos y de las evaluaciones especiales, formularios de consentimiento informado firmado, cartas del especialista y diarios de selección e inclusión de los sujetos. El investigador debe mantener archivadas estas dos clases de documentos durante, al menos, 15 años a partir

de la fecha de finalización o terminación prematura del estudio. Después de este período de tiempo, se podrán destruir los documentos, según el reglamento local.

Si el Investigator desea asignar los archivos del estudio a otra persona o trasladarlos a otro lugar, deberá notificarlo al promotor con antelación.

Si el Investigador no puede garantizar los requisitos establecidos para el archivo de la documentación en el centro de investigación, de cualquier o de todos los documentos, el Investigador y el Promotor deberán acordar medidas especiales para conservar estos documentos en una caja precintada fuera del centro, para que se puedan devolver al investigador de esta manera, en caso de una auditoría reglamentaria. Cuando se requieran los documentos originales para la asistencia continuada del paciente, se deben obtener copias apropiadas para conservar fuera del centro.

Documentos originales y datos básicos

Los investigadores deberán proporcionar al promotor, cuando lo solicite, los datos básicos requeridos de la documentación del estudio o las historias clínicas. Esto es particularmente importante cuando los CRD son ilegibles o se sospechan errores en la transcripción de los datos. En caso de que existan problemas especiales y/o de peticiones oficiales para inspecciones de auditoría, también es necesario tener acceso a todos los registros del estudio, siempre y cuando se proteja la confidencialidad del paciente.

Cuadernos de recogida de datos

Los investigadores principales o un representante autorizado del equipo del estudio, deben completar y firmar un CRD para cada paciente incluido en el estudio. Esto también se aplica a los registros de los pacientes que no completan el estudio (incluso durante el período de selección previo a la randomización, si se ha iniciado un CRD). Si un paciente se retira del estudio, se deberá indicar el motivo en el CRD. Si un paciente se retira del estudio debido a un acontecimiento adverso limitante del tratamiento, se debe intentar todo lo posible para documentar claramente el resultado.

Todos los cuadernos se deben completar a máquina o con bolígrafo de tinta negra y deben ser legibles. Los errores se deben tachar, pero no borrar, se insertará la corrección y el investigador o su representante autorizado indicará sus iniciales y la fecha en la que se ha realizado el cambio. El investigador debe asegurar que los datos comunicados al promotor en los CRD y en todos los informes requeridos son exactos, completos, legibles y puntuales.

12.- CONSIDERACIONES PRÁCTICAS

12.1.- Actuaciones tras inclusión en el ensayo y durante el seguimiento

- Debe evitarse, en lo posible, utilizar tratamiento antirretroviral con zidovudina y didanosina. En caso de fueran imprescindibles, se ajustarán las dosis a criterio del médico responsable de cada paciente.

- En mujeres en edad fértil y que puedan quedar embarazadas se realizará un test de embarazo en orina antes de iniciar tratamiento y en las semanas 4, 12, 24 y 36 (las 2 última determinación sólo en aquellas que mantengan tratamiento durante este tiempo).

- Los pacientes iniciarán tratamiento con Interferón pegilado-α 2a (135 µg/ semana) y Ribavirina (400 mg/12 horas) En aquellos pacientes con un peso < 65 kg se recomienda la administración de 300 mg/12 horas de ribavirina, aunque queda a elección del médico responsable de cada paciente. La dosificación posterior de ambos fármacos podrá disminuirse o administrarse eritopoyetina y/o GSC-F a criterio del médico responsable de cada paciente si aparecieran efectos adversos. Si es posible, la dosificación de ambos fármacos no se deben disminuir ni suprimir. En caso necesario, el médico responsable de cada paciente ajustará de forma descendente la/s dosis de Peginterferón-α 2 (ajuste de un nivel: 90 µg/semana, ajuste de dos niveles: 45 µg/semana o suspensión de la medicación) en función de la severidad de las reaciones adversas y su posible o cierta relación con la medicación en estudio). En el caso de ribavirina, se recomienda una reducción de dosis (≥ 25% de la dosis) en caso de disminución de la hemoglobina a ≤ 8,5 g/dl, sin que ello suponga obstáculo alguno para la utilización de otras medidas (eritropoyetina, transfusión de hematies) si el médico responsable del paciente así lo considera oportuno. Una vez que se hayan disminuido las dosis de Peginterferón α-2a o de ribavirina al paciente, el investigador puede intentar un nuevo aumento de la/s dosis o bien volver a administrar la/s dosis que se habian asignado originalmente al paciente, siempre y cuando el efecto adverso responsable de la disminución de la/s dosis se haya resuelto o mejorado. En caso de anemia que requiera modificación de dosis o suspensión de ribavirina debe investigarse adicionalmente metabolismo de hierro, B12 y ácido fólico en plasma.

- Se obtendrán muestras de sangre basal y en las semanas 1, 2, 4, 8 y 12 de tratamiento para los controles analíticos habituales en el seguimiento de éstos pacientes (hemograma, bioquimica, RNA-VHC, CD4 y RNA-VIH). En caso de continuar tratamiento, se obtendrán muestras en las semanas 24 y 36 (estas visitas y los controles analíticos en cada una de ellas son las habituales en el seguimiento de éstos pacientes). Deben sincronizarse las fechas de los controles analíticos con la administración semanal de interferón-α pegilado y obtenerlos tras 12 h de haber tomado la ribavirina, de forma que las muestras para concentraciones plasmáticas representen concentraciones valle de ambos fármacos.

- Para la medición de las concentraciones plasmáticas de IFN-α y ribavirina se obtendrán muestras de sangre (2 x 5 c.c. en tubos de tapón amarillo) que se centrifugará (x3000, 10 min.); el suero sobrenadante se distribuirá en criotubos, debidamente identificados por tres números (nº asignado al hospital participante / nº asignado al paciente incluido / semana de tratamiento) previo a su congelación a -20º C hasta su procesamiento mediante técnica de ELISA y HPLC, respectivamente.

- Para la determinación de ribavirina y sus metabolitos fosforilados anivel intracelular se utilizaran células mononucleares de sangre periférica obtenidas mediante gradientes de centrifugación con tubos Ficoll-Paque™. Las alícuotas serán pesadas y su volumen calculado (volumen = peso/densidad. Puesto que la densidad de estas células es 1.077 y la del plasma 1.030, el volumen de las alícuotas se igualará con su peso: Las muestras se congelarán en nitrógeno liquido hasta la determinación de las concentraciones.

- El aclaramiento de creatinina se caculará utilizando la formula de Crockcroft-Gault (concentración sérica de creatinina en mg/dL):

$$\text{Clearance de creatinina} = \frac{(140 - \text{edad en años}) \times \text{peso en kg}}{72 \times \text{creatininemia en mg/dl}}$$ Multiplicar por 0,85 si se trata de una mujer

- En función de los resultados de RNA-VHC en la semana 4 y 12 de tratamiento, los pacientes continuarán o no tratamiento como se describe en el diagrama siguiente:

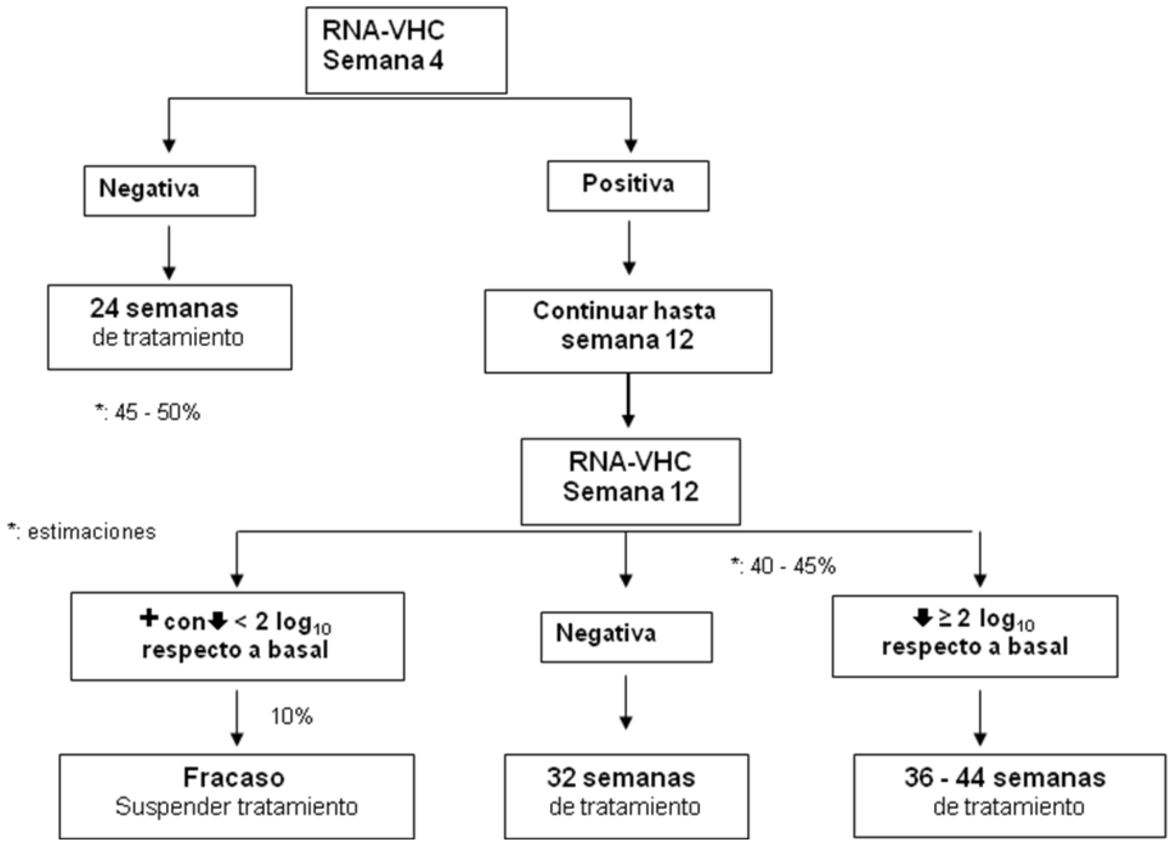

#:Aquellos pacientes con viremia positiva en la semana 4 y negativa en la semana 12 recibirán 20 semanas adicionales de tratamiento hasta completar 32 semanas. Aquellos otros con viremia positiva pero que haya disminuido ≥ 2 $\log_{10}$ respecto a la basal, en los que es de esperar una viremia negativa en las 4 semanas siguientes, recibiran un total de 36 a 44 semanas de tratamiento, de forma que todos los pacientes recibiran 20 semanas de tratamiento tras negativización de la viremia VHC medida por PCR cualitativa.

El ensayo contará con un **Comité de seguridad** compuesto por los investigadores principales en cada centro donde se lleve a cabo el ensayo clínico que realizará un análisis de los datos de eficacia y seguridad en los siguientes puntos: tras completar 12 semanas de tratamiento, fin de tratamiento y fin de seguimiento (24 semanas tras la finalización del tratamiento de la mitad de los pacientes a incluir (36 pacientes).

Procedimiento de trabajo del Comité de Seguridad: cuando la mitad de los pacientes previstos en la inclusión (36 pacientes) hayan completado 12 semanas de tratamiento y se disponga de los resultados virológicos de respuesta, el promotor e investigador coordinador enviará un informe a cada investigador principal de los centros donde se lleve a cabo el ensayo clínico. Con esta información, el Comité se reunirá y valorará dichos resultados. Sólo en caso de que la tasa de respuestas virales tempranas (definidas como RNA-VHC plasmático negativa o una disminución $\geq 2 \log_{10}$ UI/mL respecto a la basal) sea inferior al 75%, se suspenderá el ensayo clínico. Este mismo procedimiento se realizará cuando se disponga de los resultados de respuesta viral sostenida (definida como RNA-VHC plasmático negatico 24 semanas tras la finalización del tratamiento). Sólo en caso de que el porcentaje de respuestas sostenidas sea inferior al 50% se suspenderá el ensayo clínico. En ambos casos se evaluarán también los efectos adversos pero dado que se utilizarán dosis inferiores de Peginterferón a las recomendadas en la práctica clínica habitual y de Ribavirina iguales o inferiores a las recomendadas, no se espera que ello sea un motivo para interrumpir el ensayo clínico.

12.2.- Dispensación de la medicación del estudio

Toda la medicación del estudio se dispensará por los Servicios de Farmacia hospitalaria de los centros participantes con cargo a éstos ya que consiste en los fármacos habituales en el tratamiento de estos pacientes siendo especialidades farmacéuticas autorizadas y comercializadas en España para esta indicación y la decisión de tratamiento no viene condicionada por la inclusión de los pacientes en el ensayo clínico.

12.3.- Evaluación de la cumplimentación

Se pedirá a los pacientes que devuelvan en cada visita los envases utilizados y no utilizados. El personal del centro del estudio realizará un recuento de los envases y comprimidos devueltos y los verificará cotejándolos con el número entregado.

12.4.- Medicación y tratamiento concomitante

Se debe comunicar toda la medicación concomitante. Además, las dosis y la duración del tratamiento antirretroviral, en su caso, también se recogerá en el cuaderno de recogida de datos.

13.- ACONTECIMIENTOS ADVERSOS Y ANOMALÍAS DE LABORATORIO

Acontecimientos adversos clínicos

Todos los acontecimientos adversos (AA) observados durante el estudio clínico se comunicarán en el CRD. Un AA es cualquier cambio adverso desde la condición basal (pretratamiento) del paciente, incluyendo enfermedad intercurrente, que se manifieste durante un estudio clínico después de iniciar el tratamiento, se considere o no relacionado con el mismo. El "tratamiento" incluye todos los agentes experimentales (incluyendo los agentes comparativos) administrados durante el estudio.

Los AA se clasificarán en una escala de tres puntos (leve, moderado, severo; véase Anexo 4) y se comunicarán detalladamente, según se indica en el CRD. Se debe evaluar la relación del AA con el tratamiento. Véase Anexo 4.

Reacción adversa (RA) es toda reacción nociva y no intencionada a un medicamento en investigación, independientemente de la dosis administrada.

Acontecimiento adverso grave (AAG) es cualquier acontecimiento adverso que, a cualquier dosis, produzca la muerte, amenace la vida del sujeto, haga necesaria la hospitalización o la prolongación de ésta, produzca invalidez o incapacidad permanente o importante, o dé lugar a una anomalía o malformación congénita.

Se entiende por amenaza de vida la situación en que, en opinión del médico, de no haber mediado una intervención terapéutica oportuna, se hubiera producido el fallecimiento del paciente.

La hospitalización o la prolongación de una hospitalización constituye un criterio para considerar que un AA es grave. Únicamente se debe considerar como hospitalización el ingreso en el que el paciente pernocta en el hospital.

Las alteraciones de laboratorio de Grado IV serán consideradas AAG.

Reacción adversa inesperada (RAI) es aquella reacción adversa cuya naturaleza o gravedad no se corresponde con la información referente al producto (por ejemplo, el manual del investigador en el caso de un medicamento en investigación no autorizado para su comercialización, o la ficha técnica del producto en el caso de un medicamento autorizado).

En todos los acontecimientos adversos seguirán los criterios de notificación de reacciones adversas graves e inesperadas a la Agencia Española del Medicamento (anexo 5), Comités Éticos y Comunidades Autónimas, según lo establecido en el Real Decreto 223/2004 y en los apartados 22-24 del Documento de **Aclaraciones sobre la Aplicación de la Normativa de Ensayos Clínicos, versión 3.**

En todos los acontecimientos adversos graves se valorará la relación causal con el tratamiento en investigación: cuando esta relación causal se considere que existe, la reacción adversa deberá valorarse como esperada o no esperada en relación a las fichas técnicas, y se seguirán los criterios de notificación de reacciones adversas graves e inesperadas a la Agencia Española del Medicamento (anexo 4), Comités Éticos y Comunidades Autónimas, según lo establecido en el Real Decreto 223/2004 y en los apartados 22-24 del Documento de Aclaraciones sobre la Aplicación de la Normativa de Ensayos Clínicos, versión 3.

Anomalías de las pruebas de laboratorio

Los resultados de las pruebas de laboratorio se registrarán en las páginas de resultados de laboratorio del Cuaderno de Recogida de Datos. Los valores anormales de las pruebas de laboratorio, como tales, no se deben comunicar en la página de "Acontecimientos Adversos" del CRD como acontecimientos adversos, a menos que den lugar a una condición clínicamente relevante.

Manejo de los parámetros de seguridad

Acontecimientos adversos graves (que se deben comunicar inmediatamente al promotor)

El investigador debe comunicar al monitor, en el plazo de un día laborable (INMEDIATAMENTE), cualquier acontecimiento adverso que se considere GRAVE.

Todos los informes se deben enviar al Promotor del ensayo.

Un acontecimiento adverso grave es cualquier experiencia que sugiera un riesgo, contraindicación, efecto secundario o precaución significativos. Con respecto a la experiencia clínica en humanos, esto incluye cualquier experiencia que:

- es fatal o amenazante para la vida;
- requiere o prolonga la hospitalización del paciente;
- da lugar a discapacidad/incapacidad persistente o significativa;
- es una anomalía congénita/defecto de nacimiento;
- es clínicamente significativa o requiere intervención para prevenir cualquiera de los resultados indicados anteriormente.

Se debe proceder con criterio clínico y científico al decidir si es apropiada la comunicación inmediata en otras situaciones, tales como acontecimientos clínicos importantes que pueden no ser inmediatamente amenazantes para la vida o dar lugar a muerte u hospitalización, pero que pueden poner en peligro al paciente o requerir una intervención para prevenir uno de los resultados indicados anteriormente. Estas situaciones también se deben considerar normalmente graves.

El término severo es una medida de intensidad, por consiguiente, un acontecimiento adverso severo no es necesariamente grave. Por ejemplo, las náuseas de varias horas de duración se pueden clasificar como severas, pero pueden no ser clínicamente graves.

Se deben comunicar las muertes producidas durante el estudio o de las que tenga conocimiento el investigador durante las 4 semanas siguientes a la suspensión del tratamiento o durante las 24 semanas del período de seguimiento definido en el protocolo, se consideren o no relacionadas con el tratamiento.

Estos informes preliminares irán seguidos posteriormente de descripciones detalladas, que incluirán copias de las historias del hospital, informes de autopsia y otros documentos, cuando se soliciten y sea pertinente.

Para los AA graves y de otro tipo, se debe evaluar y registrar lo siguiente en la página de AA del CRD: intensidad, relación con el fármaco del estudio, medidas adoptadas en relación con el fármaco del estudio y resultado hasta la fecha.

El investigador debe notificar por escrito al Comité de Investigación Clínica (CIC) [Comité Etico Independiente (CEI)] tales acontecimientos, en cuanto sea posible y de acuerdo con las leyes y reglamento internacional y local.

Tratamiento y seguimiento de los acontecimientos adversos

Se debe realizar un seguimiento de los acontecimientos adversos, especialmente los que “no están relacionados” con la medicación del estudio, hasta que retornen al estado basal o se estabilicen. Si se establece una explicación clara, se debe registrar en el CRD. Si se considera necesario el uso de las medicaciones excluidas en el protocolo, puede que el paciente deba suspender el régimen de tratamiento del estudio, después de consultar con el coordinador.

Seguimiento de los valores anormales de las pruebas de laboratorio

En caso de que los valores de las pruebas de laboratorio muestren anomalías inexplicables, se deben repetir inmediatamente las pruebas y realizar un seguimiento hasta que los valores retornen al rango de normalidad y/o se encuentre una explicación adecuada a la anomalía. Si se establece una explicación clara, se debe registrar en el CRD.

14.- INFORMES DE SEGUIMIENTO Y FINAL Y COMUNICACIÓN DE LOS RESULTADOS

El promotor e investigador principal se compromete a presentar los informes de seguimiento y final, en los plazos establecidos y comunicar, en su caso, la interrupción y las razones de la misma (anexo2). Los resultados de este estudio serán analizados y pueden ser publicados o divulgados en revistas médicas y/o reuniones científicas. Las publicaciones que se generen serán presentadas a un comité de redacción del estudio, del que formarán parte también los promotores y el resto de investigadores involucrados en el estudio. Puesto que las revistas médicas principales restringen, en algunos casos, el número de autores la selección de los investigadores se basará en la tasa de reclutamiento.

15.- GARANTÍA DE CALIDAD

El protocolo y los Anexos relacionados y todos los datos son confidenciales. Para asegurar que todas las fuentes documentales necesarias están efectivamente disponibles y que el ensayo clínico se lleva a cabo en cumplimiento de los requerimientos de las Buenas Prácticas Clínicas, puede ser necesario la realización de un examen interno por personal apropiado designado por el investigador principal o una inspección ordenada por las Autoridades sanitarias por personal sujeto a secreto profesional. Los datos registrados en los CRD serán verificados por comprobaciones aleatorias de las fuentes de datos.

16.- FIRMA

He leído y revisado minuciosamente el protocolo de estudio. Habiendo leído y entendido los requisitos y las condiciones del protocolo de estudio, acepto realizar el estudio clínico según los principios internacionales de Buena Práctica Clínica y los requisitos de las autoridades reglamentarias para la verificación de los documentos originales y la auditoría/inspección del estudio.

Acepto utilizar el material del estudio, incluida la medicación, únicamente como se especifica en el protocolo.

Entiendo que los cambios realizados en el protocolo se deben presentar en forma de enmienda, previa aprobación por escrito del promotor.

Entiendo que cualquier violación del protocolo puede dar lugar a la terminación prematura del estudio.

Acepto respetar el calendario previsto.

Acepto comunicar a los monitores, en el plazo de un día laborable, cualquier acontecimiento adverso clínico grave, se considere o no relacionado con el tratamiento.

	**FECHA**	**FIRMA**
**INVESTIGADOR:**	/_______________/	/_____________________________/
**MONITOR:**	/_______________/	/_____________________________/

ANEXO 1

COMPROMISO DEL PROMOTOR E INVESTIGADOR COORDINADOR

Luis Fernando López Cortés, Doctor en Medicina, especialista en Medicina Interna, con ejercicio profesional como facultativo especialista en el Servicio de Enfermedades Infecciosas de los Hospitales Universitarios Virgen del Rocío, como promotor e investigador coordinador del ensayo clínico "Eficacia de dosis bajas de interferón pegilado alfa-2a y ribavirina en el tratamiento de la hepatopatía crónica por virus C (genotipos 2 o 3) en pacientes coinfectados por el VIH", se compromete a:

- Dirigir el estudio en cumplimiento con la Declaración de Helsinki, en su versión de 1974, revisada en 2000, con los requerimientos de las Buenas Prácticas Clínicas y con las normas y reglamentaciones pertinentes.
- Firmar el protocolo y cualquier modificación del mismo.
- Suministrar a los investigadores participantes el protocolo y la ficha técnica de los medicamentos a estudiar.
- Remitir el protocolo al Comité Autonómico de Ensayos Clínicos y a la Agencia Española del Medicamento.
- Presentar el protocolo del estudio y los informes de seguimiento y final, en los plazos establecidos y comunicar, en su caso, la interrupción y las razones de la misma.
- Entregar copia del protocolo y de los documentos que acrediten el seguimiento de los procedimientos establecidos en las presentes directrices a los responsables de las entidades proveedoras de servicios de atención a la salud donde se vaya a realizar el estudio.
- Comunicar las sospechas de reacciones adversas graves que surjan a lo largo del estudio al punto de contacto designado por el órgano competente en materia de farmacovigilancia de la Comunidad andaluza y a la Agencia Española del Medicamento
- Contribuir a difundir los resultados del estudio.
- No firmar ningún contrato que contenga cláusulas de secreto sobre eventuales resultados del estudio, ni comprometerse a no difundir los hallazgos de la investigación, evitando en todo caso poner a riesgo la integridad del estudio (por ejemplo, por la publicación de resultados parciales de un investigador o un centro).
- Archivar los documentos del estudio cumpliendo con normativa vigente.
- Cumplir con la confidencialidad de los documentos suministrados.
- Evitar incluir pacientes de otros estudios biomédicos.

En Sevilla, a 30 de enero de 2.007

Fdo.: Luis Fernando López Cortés

ANEXO 2

CONFORMIDAD DEL CAEC

Evaluación dell Comité Autonómico de Ensayos Clínicos de Andalucía (pendiente)

ANEXO 3

HOJA DE INFORMACION AL PACIENTE Y CONSENTIMIENTO INFORMADO

Antes de aceptar participar en este estudio y firmar el consentimiento informado, le rogamos que lea la siguiente información y realice cuantas preguntas aclaratorias desee.

Título: Eficacia de dosis bajas de interferón pegilado alfa-2a junto con ribavirina en el tratamiento de la hepatopatía crónica por virus C (genotipos 2 o 3) en pacientes coinfectados por el VIH.

¿Porqué se le pide su participación?: Actualmente el mejor tratamiento disponible para el tratamiento de la hepatopatía crónica por virus C consiste en la combinación de 2 fármacos (interferón pegilado y ribavirina). El tratamiento es eficaz para erradicar la infección por el virus C de la hepatitis en un 60 a 70% de los que finalizan el tratamiento pero no existen datos definitivos sobre la mejor dosificación o duración del tratamiento para conseguir la máxima eficacia. En este ensayo clínico queremos evaluar la eficacia de dosis algo inferiores y una duración de tratamiento algo inferior a las habitualmente utilizada ya que hay datos cientificos que apoyan una eficacia similar en el tipo de virus C que Ud. Tiene (genotipo 2 o 3) y los posibles efectos secundarios son más frecuentes e intensos con las dosis más elevadas.

¿Qué se le pide?: Su médico y Ud. han tomado la decisión de iniciar tratamiento con interferón pegilado alfa-2a y ribavirina para la hepatopatía crónica por virus C. Si acepta participar en el ensayo, la dosis de interferón que se le administrará será algo inferior a la habitual (135 µg/semana en lugar de 180 µg/semana) y la de ribavirina puede ser algo inferior si Ud. pesa menos de 65 kg (600 mg al día en lugar de 800 mg al día). El seguimiento y las extracciones analíticas serán identicos a los que se realizarían si Ud. no participa en el ensayo clínico excepto que en cada extracción se obtendrá 1 tubo más para medir la cantidad de antibiótico que hay en su sangre para analizar si hay relación con el resultado final del tratamiento.

¿Existen riesgos por participar en el ensayo?. Creemos que los posibles efectos adversos derivados de la medicación serán menos frecuentes y/o de menor intensidad con las dosis que le proponemos que con las dosis habituales. El único riesgo sería una eficacia algo menor del tratamiento aunque estamos convencidos de que no será así y eso es lo que queremos probar.

El ensayo clínico cuenta con la aprobación de las Autoridades Sanitarias correspondientes (Comités Éticos de todos los hospitales participantes, Comité Autonómico de Andalucía de Ensayos Clínicos y del Ministerio de Sanidad y Consumo - Agencia Española del Medicamento y Productos Sanitarios) y todos los requisitos legales vigentes de acuerdo con Real Decreto 223/2004, de 6 de febrero de 2004, por el que se regulan los ensayos clínicos con medicamentos (BOE núm. 33, de 7 febrero de 2004), incluyendo un seguro de responsabilidad civil por parte del promotor y los investigadores.

Confidencialidad de los datos: Los datos del estudio se transcribirán a formularios de recogida de datos en los que sólo figurarán las iniciales de los pacientes y un código asignado para cada paciente, que servirá de para la identificación de pacientes y muestras de sangre según la Ley 15/1999, de 13 de diciembre de Protección de Datos Personales.

Únicamente los médicos y enfermeros(as) participantes en el estudio y el personal autorizado por los organismos oficiales, en caso necesario, podrán tener acceso a la historia clínica de las pacientes.

Si usted es mujer en edad fértil, no debe estar embarazada y debe utilizar un método anticonceptivo seguro hasta al menos 6 meses después de haber finalizado la medicación.

Yo (nombre y apellidos) :

He leído la hoja de información que se me ha entregado y he podido hacer preguntas sobre el estudio.

He hablado con (nombre del investigador) ....

Comprendo que mi participación es voluntaria y presto libremente mi conformidad para participar en el ensayo, puediendo retirarse libremente, sin necesidad de dar explicaciones, y sin que ello afecte en la relación con el/los médico/s que me atienden.

Fecha   Firma del participante

Para cualquier duda o problema relacionado con el estudio podrá usted dirigirse a su médico,

el Dr.: ....

Teléfono: ....

ANEXO 4

Anexo D Formulario de notificación de reacción adversa grave e inesperada ocurrida en España

NOTIFICACION DE SOSPECHA DE REACCION ADVERSA PARA MEDICAMENTOS EN INVESTIGACIÓN	CODIGO DE PROTOCOLO (promotor)………………. Nº EUDRACT/ Nº Protocolo AEMPS………………..	Nº NOTIFICACION (Promotor)
Notificación realizada a Eudravigilance ☐ SI ☐ NO	PACIENTE Nº	Nº NOTIFICACION

INFORMACION SOBRE LA REACCIÓN ADVERSA

1a. PAÍS	2. FECHA DE NACIMIENTO			2a. EDAD	3. SEXO	3a. PESO	3b. TALLA	4-6. FECHA DE INICIO DE LA REACCIÓN		
	DÍA	MES	AÑO		☐ HOMBRE<br>☐ MUJER			DÍA	MES	AÑO

7. DESCRIPCIÓN DE LA REACCIÓN ADVERSA (Incluyendo resultados relevantes de exploración o de laboratorio, y la fecha de finalización, si procede).	8-13b. CRITERIOS DE GRAVEDAD/ DESENLACE<br><br>☐ FALLECIMIENTO<br>☐ LA VIDA DEL PACIENTE HA ESTADO EN PELIGRO<br>☐ HOSPITALIZACIÓN<br>☐ PROLONGACIÓN HOSPITALIZACIÓN<br>☐ **INCAPACIDAD PERMANENTE O SIGNIFICATIVA**<br>☐ **RA CLINICAMENTE RELEVANTE**<br>**Desenlace**<br>☐ PERSISTENCIA DE LA RA<br>☐ RECUPERACIÓN SIN SECUELAS<br>☐ RECUPERACIÓN CON SECUELAS<br>☐ DESCONOCIDO

II. INFORMACION DEL MEDICAMENTO EN INVESTIGACIÓN

14. MEDICAMENTO SOSPECHOSO	15. DOSIS DIARIA	16. VÍA	17. ENFERMEDAD EN ESTUDIO	18. FECHAS DE INICIO	FINAL	19. DURACIÓN DEL TRATAMIENTO

20. ¿REMITIÓ LA REACCIÓN AL SUSPENDER LA MEDICACIÓN? ☐ SI ☐ NO ☐ NO PROCEDE	20a. ¿REMITIÓ LA REACCIÓN AL REDUCIR LA DOSIS? ☐ SI ☐ NO ☐ NO PROCEDE	21. ¿REAPARECIÓ LA REACCIÓN AL ADMINISTRAR DE NUEVO LA MEDICACIÓN? ☐ SI ☐ NO ☐ NO PROCEDE

III. MEDICAMENTOS CONCOMITANTES E HISTORIA CLÍNICA

22. MEDICAMENTOS CONCOMITANTES (Márquese con un asterisco el o los medicamentos sospechosos)	22a. DOSIS DIARIA	22b. VÍA	22c. FECHAS DE INICIO	FINAL	22d. MOTIVO DE LA PRESCRIPCIÓN
23. DATOS IMPORTANTES DE LA HISTORIA CLÍNICA (ej. diagnósticos, alergias, embarazos, etc.)					

IV. INFORMACION SOBRE PROMOTOR E INVESTIGADOR

24a. NOMBRE Y DIRECCION DEL PROMOTOR		24b. NOMBRE Y DIRECCION DEL INVESTIGADOR
24c. CODIGO DE LABORATORIO (Nº AEMPS)	25a. TIPO DE INFORME<br>☐ INICIAL<br>☐ SEGUIMIENTO	24c. TECNICO DEL PROMOTOR QUE INFORMA<br>NOMBRE:<br>TELEFONO:<br>FIRMA:
24e. FECHA DEL INFORME	24f. FECHA DE ENTRADA AEM	25b. SE ADJUNTA INFORME COMPLEMENTARIO

INSTRUCCIONES GENERALES

1. Este formulario se utilizará solamente para comunicar las sospechas de reacciones adversas (RA) graves e inesperadas que ocurran con medicamentos en investigación.

2. Las sospechas de RA mortales o que entrañen riesgo vital (aquellas que de no haber mediado una intervención terapéutica inmediata hubieran supuesto la muerte del paciente) se comunicarán en el plazo máximo de 7 días naturales; si no se dispusiera de toda la información, ésta podrá completarse en el plazo adicional de 8 días. Las demás sospechas de RA graves e inesperadas se comunicarán en el plazo máximo de 15 días.

3. Cuando el espacio disponible sea insuficiente, se añadirá una hoja de información adicional, correctamente identificada con el nombre del promotor y el número asignado a la notificación. En dicha información podrá hacerse constar la evaluación de la causalidad realizada por el técnico que informa.

INSTRUCCIONES ESPECÍFICAS

Ø. El código de protocolo es el asignado por el promotor para identificar el ensayo. El número de notificación del promotor es el que éste utiliza para su archivo. Cuando se trate de información de seguimiento se utilizará el mismo número o bien, si se modifica, se indicará el número de la notificación inicial. Se dejará sin rellenar el espacio “Nº de notificación” que aparece sombreado.

2. La edad se pondrá en años, meses, semanas o días según convenga, pero siempre indicándolo. Si no se conoce con precisión la edad debe referirse, al menos, el grupo de edad al que pertenece (p. ej.: lactante, niño, adolescente, adulto, anciano).

7. Se describirá la RA en forma completa, indicando la fecha de finalización de la misma e incluyendo los resultados de las exploraciones complementarias o pruebas de laboratorio que se consideren de interés. A esta notificación podrán acompañarse cuantos informes se estimen convenientes para la adecuada interpretación del cuadro clínico sospechoso de ser una reacción adversa.

8-13. Las categorías no son mutuamente excluyentes. La asistencia en un Servicio de Urgencias de un Hospital inferior a 24 horas, no se considerará hospitalización.

14. Los medicamentos en investigación se identificarán a ser posible por su nombre genérico (DOE o DCI), indicando cuando esté disponible el nombre comercial, o en su defecto, por el nombre propuesto o código de laboratorio para el producto.

15. En caso de que la administración no sea diaria se intentará describirla con alguna de las siguientes posibilidades: cíclica, semanal, mensual, anual o número de veces que se ha utilizado (poniendo en este caso la dosis de cada toma, no la total).

17. Se hará constar el proceso patológico del paciente al que va destinado el producto en investigación, o bien “voluntario sano” en caso de tratarse de tal.

19. Se hará constar la duración del tratamiento hasta el inicio de la reacción adversa.

22. Se indicará explícitamente si no se han tomado fármacos concomitantes. En el caso de considerar sospechoso alguno o algunos de los fármacos concomitantes se marcarán con un asterisco (p.ej.: * AMOXICILINA). Se excluirán los medicamentos utilizados para tratar la reacción adversa.

Acontecimientos adversos - Relación con la medicación del ensayo

PROBABLE (se deben manifestar las tres primeras)

Esta categoría se aplica a los acontecimientos adversos que se consideran, con un grado alto de seguridad, relacionados con la medicación del ensayo. Un acontecimiento adverso se puede considerar probable si:

1. Sigue una secuencia temporal razonable desde la administración del fármaco.
2. No se puede explicar de manera razonable por las características conocidas del estado clínico del paciente, factores ambientales o tóxicos, u otro tipo de terapia administrada al paciente.
3. Desaparece o disminuye al suspender o reducir la dosis (Existen importantes excepciones cuando un acontecimiento adverso no desaparece al suspender la medicación, sin embargo, existe una relación clara con el fármaco, ej (1) mielosupresión, (2) discinesias tardías.)
4. Sigue un patrón de respuesta conocido al fármaco sospechoso.
5. Reaparece con una nueva exposición al fármaco.

POSIBLE (se deben manifestar las dos primeras)

Esta categoría se aplica a los acontecimientos adversos cuya relación con la administración de la medicación del estudio parece poco probable, pero no se puede descartar con seguridad. Un acontecimiento adverso se puede considerar posible si o cuando:

1. Sigue una secuencia temporal razonable desde la administración del fármaco.
2. Se podría haber producido por el estado clínico del paciente, factores ambientales o tóxicos o el tipo de terapia administrada al paciente.
3. Sigue un patrón de respuesta conocido al fármaco sospechoso.

REMOTA (se deben manifestar las dos primeras)

En general, esta categoría es aplicable a un acontecimiento adverso que cumpla los criterios siguientes:

1. No sigue una secuencia temporal razonable desde la administración del fármaco.
2. Se podría haber producido fácilmente por el estado clínico del paciente, factores ambientales o tóxicos u otro tipo de terapia administrada al paciente.
3. No sigue un patrón de respuesta conocido al fármaco sospechoso.
4. No reaparece ni se empeora cuando se vuelve a administrar el fármaco.

NO RELACIONADA

Esta categoría es aplicable a los acontecimientos adversos que, después de un razonamiento médico minucioso en el momento de la evaluación, se consideran clara e indiscutiblemente debidos sólo a causas ajenas (enfermedad, entorno, etc) y no cumplen los criterios de la relación con la medicación descritos anteriormente en las categorías de REMOTA, POSIBLE O PROBABLE.

Categorías de los acontecimientos adversos - Relación esquemática con la medicación del estudio

	Probable	Posible	Remota	No relacionado
Claramente debidos a causas ajenas	-	-	-	+
Asociación temporal razonable con la administración del fármaco	+	+	-	-
Puede estar provocado por el estado clínico del sujeto, etc.	-	+	+	+
Patrón de respuesta conocido al fármaco sospechoso	+	+	-	-
Desaparece o disminuye al suspender o reducir la dosis	+	-	-	-
Reaparece al volver a administrar el fármaco	+	-	-	-

Acontecimientos adversos - Intensidad

La intensidad (leve, moderada, severa) de los acontecimientos adversos clínicos se debe determinar mediante una escala apropiada y se deben definir claramente los términos utilizados en la escala, como en el ejemplo siguiente:

Leve:
se observan molestias, pero no alteran la actividad normal cotidiana

Moderado:
molestias sufucientes para reducir o afectar a la actividad normal cotidiana

Severo:
discapacitante para poder trabajar o desempeñar las actividades normales cotidianas

DIVISION OF AIDS TABLE FOR GRADING THE SEVERITY OF ADULT AND PEDIATRIC ADVERSE EVENTS PUBLISH DATE: DECEMBER, 2004

CLINICAL				
**PARAMETER**	**GRADE 1 MILD**	**GRADE 2 MODERATE**	**GRADE 3 SEVERE**	**GRADE 4 POTENTIALLY LIFE-THREATENING**
**ESTIMATING SEVERITY GRADE**				
Clinical adverse event NOT identified elsewhere in this DAIDS AE grading table	Symptoms causing no or minimal interference with usual social & functional activities	Symptoms causing greater than minimal interference with usual social & functional activities	Symptoms causing inability to perform usual social & functional activities	Symptoms causing inability to perform basic self-care functions OR Medical or operative intervention indicated to prevent permanent impairment, persistent disability, or death
**SYSTEMIC**				
Acute systemic allergic reaction	Localized urticaria (wheals) with no medical intervention indicated	Localized urticaria with medical intervention indicated OR Mild angioedema with no medical intervention indicated	Generalized urticaria OR Angioedema with medical intervention indicated OR Symptomatic mild bronchospasm	Acute anaphylaxis OR Life-threatening bronchospasm OR laryngeal edema
Chills	Symptoms causing no or minimal interference with usual social & functional activities	Symptoms causing greater than minimal interference with usual social & functional activities	Symptoms causing inability to perform usual social & functional activities	NA
Fatigue<br>Malaise	Symptoms causing no or minimal interference with usual social & functional activities	Symptoms causing greater than minimal interference with usual social & functional activities	Symptoms causing inability to perform usual social & functional activities	Incapacitating fatigue/ malaise symptoms causing inability to perform basic self-care functions
Fever (nonaxillary)	37.7 – 38.6°C	38.7 – 39.3°C	39.4 – 40.5°C	> 40.5°C
Pain (indicate body site)<br>DO NOT use for pain due to injection (See Injection Site Reactions: Injection site pain)<br>See also Headache, Arthralgia, and Myalgia	Pain causing no or minimal interference with usual social & functional activities	Pain causing greater than minimal interference with usual social & functional activities	Pain causing inability to perform usual social & functional activities	Disabling pain causing inability to perform basic self-care functions OR Hospitalization (other than emergency room visit) indicated
Unintentional weight loss	NA	5 – 9% loss in body weight from baseline	10 – 19% loss in body weight from baseline	≥ 20% loss in body weight from baseline OR Aggressive intervention indicated [e.g., tube feeding or total parenteral nutrition (TPN)]

CLINICAL				
**PARAMETER**	**GRADE 1 MILD**	**GRADE 2 MODERATE**	**GRADE 3 SEVERE**	**GRADE 4 POTENTIALLY LIFE-THREATENING**
**INFECTION**				
Infection (any other than HIV infection)	Localized, no systemic antimicrobial treatment indicated AND Symptoms causing no or minimal interference with usual social & functional activities	Systemic antimicrobial treatment indicated OR Symptoms causing greater than minimal interference with usual social & functional activities	Systemic antimicrobial treatment indicated AND Symptoms causing inability to perform usual social & functional activities OR Operative intervention (other than simple incision and drainage) indicated	Life-threatening consequences (e.g., septic shock)
**INJECTION SITE REACTIONS**				
Injection site pain (pain without touching) Or Tenderness (pain when area is touched)	Pain/tenderness causing no or minimal limitation of use of limb	Pain/tenderness limiting use of limb OR Pain/tenderness causing greater than minimal interference with usual social & functional activities	Pain/tenderness causing inability to perform usual social & functional activities	Pain/tenderness causing inability to perform basic self-care function OR Hospitalization (other than emergency room visit) indicated for management of pain/tenderness
Injection site reaction (localized)				
**Adult > 15 years**	Erythema OR Induration of 5x5 cm – 9x9 cm (or 25 cm^2 – 81cm^2)	Erythema OR Induration OR Edema > 9 cm any diameter (or > 81 cm^2)	Ulceration OR Secondary infection OR Phlebitis OR Sterile abscess OR Drainage	Necrosis (involving dermis and deeper tissue)
**SKIN – DERMATOLOGICAL**				
Pruritis associated with injection See also Skin: Pruritis (itching - no skin lesions)	Itching localized to injection site AND Relieved spontaneously or with < 48 hours treatment	Itching beyond the injection site but not generalized OR Itching localized to injection site requiring ≥ 48 hours treatment	Generalized itching causing inability to perform usual social & functional activities	NA
Alopecia	Thinning detectable by study participant (or by caregiver for young children and disabled adults)	Thinning or patchy hair loss detectable by health care provider	Complete hair loss	NA
Cutaneous reaction – rash	Localized macular rash	Diffuse macular, maculopapular, or morbilliform rash OR Target lesions	Diffuse macular, maculopapular, or morbilliform rash with vesicles or limited number of bullae OR Superficial ulcerations of mucous membrane limited to one site	Extensive or generalized bullous lesions OR Stevens-Johnson syndrome OR Ulceration of mucous membrane involving two or more distinct mucosal sites OR Toxic epidermal necrolysis (TEN)
Hyperpigmentation	Slight or localized	Marked or generalized	NA	NA
Hypopigmentation	Slight or localized	Marked or generalized	NA	NA

CLINICAL				
**PARAMETER**	**GRADE 1 MILD**	**GRADE 2 MODERATE**	**GRADE 3 SEVERE**	**GRADE 4 POTENTIALLY LIFE-THREATENING**
Pruritis (itching – no skin lesions)<br>(See also Injection Site Reactions: Pruritis associated with injection)	Itching causing no or minimal interference with usual social & functional activities	Itching causing greater than minimal interference with usual social & functional activities	Itching causing inability to perform usual social & functional activities	NA
**CARDIOVASCULAR**				
Cardiac arrhythmia (general)<br>(By ECG or physical exam)	Asymptomatic AND No intervention indicated	Asymptomatic AND Non-urgent medical intervention indicated	Symptomatic, non-life-threatening AND Non-urgent medical intervention indicated	Life-threatening arrhythmia OR Urgent intervention indicated
Cardiac-ischemia/infarction	NA	NA	Symptomatic ischemia (stable angina) OR Testing consistent with ischemia	Unstable angina OR Acute myocardial infarction
Hemorrhage (significant acute blood loss)	NA	Symptomatic AND No transfusion indicated	Symptomatic AND Transfusion of ≤ 2 units packed RBCs (for children ≤ 10 cc/kg) indicated	Life-threatening hypotension OR Transfusion of > 2 units packed RBCs (for children > 10 cc/kg) indicated
Hypertension				
**Adult > 17 years**<br>(with repeat testing at same visit)	> 140 – 159 mmHg systolic<br>OR<br>> 90 – 99 mmHg diastolic	> 160 – 179 mmHg systolic<br>OR<br>> 100 – 109 mmHg diastolic	> 180 mmHg systolic<br>OR<br>> 110 mmHg diastolic	Life-threatening consequences (e.g., malignant hypertension) OR Hospitalization indicated (other than emergency room visit)
Hypotension	NA	Symptomatic, corrected with oral fluid replacement	Symptomatic, IV fluids indicated	Shock requiring use of vasopressors or mechanical assistance to maintain blood pressure
Pericardial effusion	Asymptomatic, small effusion requiring no intervention	Asymptomatic, moderate or larger effusion requiring no intervention	Effusion with non-life threatening physiologic consequences OR Effusion with non-urgent intervention indicated	Life-threatening consequences (e.g., tamponade) OR Urgent intervention indicated
Prolonged PR interval				
**Adult > 16 years**	PR interval 0.21 – 0.25 sec	PR interval > 0.25 sec	Type II 2nd degree AV block OR Ventricular pause > 3.0 sec	Complete AV block
Prolonged QTc				
**Adult > 16 years**	Asymptomatic, QTc interval 0.45 – 0.47 sec OR Increase interval < 0.03 sec above baseline	Asymptomatic, QTc interval 0.48 – 0.49 sec OR Increase in interval 0.03 – 0.05 sec above baseline	Asymptomatic, QTc interval ≥ 0.50 sec OR Increase in interval ≥ 0.06 sec above baseline	Life-threatening consequences, e.g. Torsade de pointes or other associated serious ventricular dysrhythmia

CLINICAL				
**PARAMETER**	**GRADE 1 MILD**	**GRADE 2 MODERATE**	**GRADE 3 SEVERE**	**GRADE 4 POTENTIALLY LIFE-THREATENING**
Thrombosis/embolism	NA	Deep vein thrombosis AND No intervention indicated (e.g., anticoagulation, lysis filter, invasive procedure)	Deep vein thrombosis AND Intervention indicated (e.g., anticoagulation, lysis filter, invasive procedure)	Embolic event (e.g., pulmonary embolism, life-threatening thrombus)
Vasovagal episode (associated with a procedure of any kind)	Present without loss of consciousness	Present with transient loss of consciousness	NA	NA
Ventricular dysfunction (congestive heart failure)	NA	Asymptomatic diagnostic finding AND intervention indicated	New onset with symptoms OR Worsening symptomatic congestive heart failure	Life-threatening congestive heart failure
**GASTROINTESTINAL**				
Anorexia	Loss of appetite without decreased oral intake	Loss of appetite associated with decreased oral intake without significant weight loss	Loss of appetite associated with significant weight loss	Life-threatening consequences OR Aggressive intervention indicated [e.g., tube feeding or total parenteral nutrition (TPN)]
Ascites	Asymptomatic	Symptomatic AND Intervention indicated (e.g., diuretics or therapeutic paracentesis)	Symptomatic despite intervention	Life-threatening consequences
Cholecystitis	NA	Symptomatic AND Medical intervention indicated	Radiologic, endoscopic, or operative intervention indicated	Life-threatening consequences (e.g., sepsis or perforation)
Constipation	NA	Persistent constipation requiring regular use of dietary modifications, laxatives, or enemas	Obstipation with manual evacuation indicated	Life-threatening consequences (e.g., obstruction)
Diarrhea				
**Adult**	Transient or intermittent episodes of unformed stools OR Increase of ≤ 3 stools over baseline per 24-hour period	Persistent episodes of unformed to watery stools OR Increase of 4 – 6 stools over baseline per 24-hour period	Bloody diarrhea OR Increase of ≥ 7 stools per 24-hour period OR IV fluid replacement indicated	Life-threatening consequences (e.g., hypotensive shock)
Dysphagia-Odynophagia	Symptomatic but able to eat usual diet	Symptoms causing altered dietary intake without medical intervention indicated	Symptoms causing severely altered dietary intake with medical intervention indicated	Life-threatening reduction in oral intake

CLINICAL				
**PARAMETER**	**GRADE 1 MILD**	**GRADE 2 MODERATE**	**GRADE 3 SEVERE**	**GRADE 4 POTENTIALLY LIFE-THREATENING**
Mucositis/stomatitis (clinical exam)<br>Indicate site (e.g., larynx, oral)<br>See Genitourinary for Vulvovaginitis<br>See also Dysphagia-Odynophagia and Proctitis	Erythema of the mucosa	Patchy pseudomembranes or ulcerations	Confluent pseudomembranes or ulcerations OR Mucosal bleeding with minor trauma	Tissue necrosis OR Diffuse spontaneous mucosal bleeding OR Life-threatening consequences (e.g., aspiration, choking)
Nausea	Transient (< 24 hours) or intermittent nausea with no or minimal interference with oral intake	Persistent nausea resulting in decreased oral intake for 24 – 48 hours	Persistent nausea resulting in minimal oral intake for > 48 hours OR Aggressive rehydration indicated (e.g., IV fluids)	Life-threatening consequences (e.g., hypotensive shock)
Pancreatitis	NA	Symptomatic AND Hospitalization not indicated (other than emergency room visit)	Symptomatic AND Hospitalization indicated (other than emergency room visit)	Life-threatening consequences (e.g., circulatory failure, hemorrhage, sepsis)
Proctitis (functional-symptomatic)<br>Also see Mucositis/stomatitis for clinical exam	Rectal discomfort AND No intervention indicated	Symptoms causing greater than minimal interference with usual social & functional activities OR Medical intervention indicated	Symptoms causing inability to perform usual social & functional activities OR Operative intervention indicated	Life-threatening consequences (e.g., perforation)
Vomiting	Transient or intermittent vomiting with no or minimal interference with oral intake	Frequent episodes of vomiting with no or mild dehydration	Persistent vomiting resulting in orthostatic hypotension OR Aggressive rehydration indicated (e.g., IV fluids)	Life-threatening consequences (e.g., hypotensive shock)
**NEUROLOGIC**				
Alteration in personality-behavior or in mood (e.g., agitation, anxiety, depression, mania, psychosis)	Alteration causing no or minimal interference with usual social & functional activities	Alteration causing greater than minimal interference with usual social & functional activities	Alteration causing inability to perform usual social & functional activities	Behavior potentially harmful to self or others (e.g., suicidal and homicidal ideation or attempt, acute psychosis) OR Causing inability to perform basic self-care functions
Altered Mental Status<br>For Dementia, see Cognitive and behavioral/attentional disturbance (including dementia and attention deficit disorder)	Changes causing no or minimal interference with usual social & functional activities	Mild lethargy or somnolence causing greater than minimal interference with usual social & functional activities	Confusion, memory impairment, lethargy, or somnolence causing inability to perform usual social & functional activities	Delirium OR obtundation, OR coma

CLINICAL				
**PARAMETER**	**GRADE 1 MILD**	**GRADE 2 MODERATE**	**GRADE 3 SEVERE**	**GRADE 4 POTENTIALLY LIFE-THREATENING**
Ataxia	Asymptomatic ataxia detectable on exam OR Minimal ataxia causing no or minimal interference with usual social & functional activities	Symptomatic ataxia causing greater than minimal interference with usual social & functional activities	Symptomatic ataxia causing inability to perform usual social & functional activities	Disabling ataxia causing inability to perform basic self-care functions
Cognitive and behavioral/attentional disturbance (including dementia and attention deficit disorder)	Disability causing no or minimal interference with usual social & functional activities OR Specialized resources not indicated	Disability causing greater than minimal interference with usual social & functional activities OR Specialized resources on part-time basis indicated	Disability causing inability to perform usual social & functional activities OR Specialized resources on a full-time basis indicated	Disability causing inability to perform basic self-care functions OR Institutionalization indicated
CNS ischemia (acute)	NA	NA	Transient ischemic attack	Cerebral vascular accident (CVA, stroke) with neurological deficit
Headache	Symptoms causing no or minimal interference with usual social & functional activities	Symptoms causing greater than minimal interference with usual social & functional activities	Symptoms causing inability to perform usual social & functional activities	Symptoms causing inability to perform basic self-care functions OR Hospitalization indicated (other than emergency room visit) OR Headache with significant impairment of alertness or other neurologic function
Insomnia	NA	Difficulty sleeping causing greater than minimal interference with usual social & functional activities	Difficulty sleeping causing inability to perform usual social & functional activities	Disabling insomnia causing inability to perform basic self-care functions
Neuromuscular weakness (including myopathy & neuropathy)	Asymptomatic with decreased strength on exam OR Minimal muscle weakness causing no or minimal interference with usual social & functional activities	Muscle weakness causing greater than minimal interference with usual social & functional activities	Muscle weakness causing inability to perform usual social & functional activities	Disabling muscle weakness causing inability to perform basic self-care functions OR Respiratory muscle weakness impairing ventilation
Neurosensory alteration (including paresthesia and painful neuropathy)	Asymptomatic with sensory alteration on exam or minimal paresthesia causing no or minimal interference with usual social & functional activities	Sensory alteration or paresthesia causing greater than minimal interference with usual social & functional activities	Sensory alteration or paresthesia causing inability to perform usual social & functional activities	Disabling sensory alteration or paresthesia causing inability to perform basic self-care functions
Seizure: (new onset) **– Adult ≥ 18 years**<br>See also Seizure: (known pre-existing seizure disorder)	NA	1 seizure	2 – 4 seizures	Seizures of any kind which are prolonged, repetitive (e.g., status epilepticus), or difficult to control (e.g., refractory epilepsy)

CLINICAL				
**PARAMETER**	**GRADE 1 MILD**	**GRADE 2 MODERATE**	**GRADE 3 SEVERE**	**GRADE 4 POTENTIALLY LIFE-THREATENING**
Seizure: (known pre-existing seizure disorder) **– Adult ≥ 18 years** For worsening of existing epilepsy the grades should be based on an increase from previous level of control to any of these levels.	NA	Increased frequency of pre-existing seizures (non-repetitive) without change in seizure character OR Infrequent break-through seizures while on stable medication in a previously controlled seizure disorder	Change in seizure character from baseline either in duration or quality (e.g., severity or focality)	Seizures of any kind which are prolonged, repetitive (e.g., status epilepticus), or difficult to control (e.g., refractory epilepsy)
Syncope (not associated with a procedure)	NA	Present	NA	NA
Vertigo	Vertigo causing no or minimal interference with usual social & functional activities	Vertigo causing greater than minimal interference with usual social & functional activities	Vertigo causing inability to perform usual social & functional activities	Disabling vertigo causing inability to perform basic self-care functions
**RESPIRATORY**				
Bronchospasm (acute)	FEV1 or peak flow reduced to 70 – 80%	FEV1 or peak flow 50 – 69%	FEV1 or peak flow 25 – 49%	Cyanosis OR FEV1 or peak flow < 25% OR Intubation
Dyspnea or respiratory distress				
**Adult ≥ 14 years**	Dyspnea on exertion with no or minimal interference with usual social & functional activities	Dyspnea on exertion causing greater than minimal interference with usual social & functional activities	Dyspnea at rest causing inability to perform usual social & functional activities	Respiratory failure with ventilatory support indicated
**MUSCULOSKELETAL**				
Arthralgia See also Arthritis	Joint pain causing no or minimal interference with usual social & functional activities	Joint pain causing greater than minimal interference with usual social & functional activities	Joint pain causing inability to perform usual social & functional activities	Disabling joint pain causing inability to perform basic self-care functions
Arthritis See also Arthralgia	Stiffness or joint swelling causing no or minimal interference with usual social & functional activities	Stiffness or joint swelling causing greater than minimal interference with usual social & functional activities	Stiffness or joint swelling causing inability to perform usual social & functional activities	Disabling joint stiffness or swelling causing inability to perform basic self-care functions
Bone Mineral Loss				
**Adults**	BMD t-score -2.5 to -1.0	BMD t-score < -2.5	Pathological fracture (including loss of vertebral height)	Pathologic fracture causing life-threatening consequences
Myalgia (non-injection site)	Muscle pain causing no or minimal interference with usual social & functional activities	Muscle pain causing greater than minimal interference with usual social & functional activities	Muscle pain causing inability to perform usual social & functional activities	Disabling muscle pain causing inability to perform basic self-care functions

CLINICAL				
**PARAMETER**	**GRADE 1 MILD**	**GRADE 2 MODERATE**	**GRADE 3 SEVERE**	**GRADE 4 POTENTIALLY LIFE-THREATENING**
Osteonecrosis	NA	Asymptomatic with radiographic findings AND No operative intervention indicated	Symptomatic bone pain with radiographic findings OR Operative intervention indicated	Disabling bone pain with radiographic findings causing inability to perform basic self-care functions
**GENITOURINARY**				
Cervicitis (symptoms)<br>(For use in studies evaluating topical study agents)<br>For other cervicitis see Infection: Infection (any other than HIV infection)	Symptoms causing no or minimal interference with usual social & functional activities	Symptoms causing greater than minimal interference with usual social & functional activities	Symptoms causing inability to perform usual social & functional activities	Symptoms causing inability to perform basic self-care functions
Cervicitis (clinical exam)<br>(For use in studies evaluating topical study agents)<br>For other cervicitis see Infection: Infection (any other than HIV infection)	Minimal cervical abnormalities on examination (erythema, mucopurulent discharge, or friability) OR Epithelial disruption < 25% of total surface	Moderate cervical abnormalities on examination (erythema, mucopurulent discharge, or friability) OR Epithelial disruption of 25 – 49% total surface	Severe cervical abnormalities on examination (erythema, mucopurulent discharge, or friability) OR Epithelial disruption 50 – 75% total surface	Epithelial disruption > 75% total surface
Inter-menstrual bleeding (IMB)	Spotting observed by participant OR Minimal blood observed during clinical or colposcopic examination	Inter-menstrual bleeding not greater in duration or amount than usual menstrual cycle	Inter-menstrual bleeding greater in duration or amount than usual menstrual cycle	Hemorrhage with life-threatening hypotension OR Operative intervention indicated
Urinary tract obstruction (e.g., stone)	NA	Signs or symptoms of urinary tract obstruction without hydronephrosis or renal dysfunction	Signs or symptoms of urinary tract obstruction with hydronephrosis or renal dysfunction	Obstruction causing life-threatening consequences
Vulvovaginitis (symptoms)<br>(Use in studies evaluating topical study agents)<br>For other vulvovaginitis see Infection: Infection (any other than HIV infection)	Symptoms causing no or minimal interference with usual social & functional activities	Symptoms causing greater than minimal interference with usual social & functional activities	Symptoms causing inability to perform usual social & functional activities	Symptoms causing inability to perform basic self-care functions

CLINICAL				
**PARAMETER**	**GRADE 1 MILD**	**GRADE 2 MODERATE**	**GRADE 3 SEVERE**	**GRADE 4 POTENTIALLY LIFE-THREATENING**
Vulvovaginitis (clinical exam)<br>(Use in studies evaluating topical study agents)<br>For other vulvovaginitis see Infection: Infection (any other than HIV infection)	Minimal vaginal abnormalities on examination OR Epithelial disruption < 25% of total surface	Moderate vaginal abnormalities on examination OR Epithelial disruption of 25 - 49% total surface	Severe vaginal abnormalities on examination OR Epithelial disruption 50 - 75% total surface	Vaginal perforation OR Epithelial disruption > 75% total surface
**OCULAR/VISUAL**				
Uveitis	Asymptomatic but detectable on exam	Symptomatic anterior uveitis OR Medical intervention indicated	Posterior or pan-uveitis OR Operative intervention indicated	Disabling visual loss in affected eye(s)
Visual changes (from baseline)	Visual changes causing no or minimal interference with usual social & functional activities	Visual changes causing greater than minimal interference with usual social & functional activities	Visual changes causing inability to perform usual social & functional activities	Disabling visual loss in affected eye(s)
**ENDOCRINE/METABOLIC**				
Abnormal fat accumulation (e.g., back of neck, breasts, abdomen)	Detectable by study participant (or by caregiver for young children and disabled adults)	Detectable on physical exam by health care provider	Disfiguring OR Obvious changes on casual visual inspection	NA
Diabetes mellitus	NA	New onset without need to initiate medication OR Modification of current medications to regain glucose control	New onset with initiation of medication indicated OR Diabetes uncontrolled despite treatment modification	Life-threatening consequences (e.g., ketoacidosis, hyperosmolar non-ketotic coma)
Gynecomastia	Detectable by study participant or caregiver (for young children and disabled adults)	Detectable on physical exam by health care provider	Disfiguring OR Obvious on casual visual inspection	NA
Hyperthyroidism	Asymptomatic	Symptomatic causing greater than minimal interference with usual social & functional activities OR Thyroid suppression therapy indicated	Symptoms causing inability to perform usual social & functional activities OR Uncontrolled despite treatment modification	Life-threatening consequences (e.g., thyroid storm)
Hypothyroidism	Asymptomatic	Symptomatic causing greater than minimal interference with usual social & functional activities OR Thyroid replacement therapy indicated	Symptoms causing inability to perform usual social & functional activities OR Uncontrolled despite treatment modification	Life-threatening consequences (e.g., myxedema coma)

CLINICAL				
**PARAMETER**	**GRADE 1<br>MILD**	**GRADE 2<br>MODERATE**	**GRADE 3<br>SEVERE**	**GRADE 4<br>POTENTIALLY LIFE-THREATENING**
Lipoatrophy<br>(e.g., fat loss from the face, extremities, buttocks)	Detectable by study participant (or by caregiver for young children and disabled adults)	Detectable on physical exam by health care provider	Disfiguring OR Obvious on casual visual inspection	NA

LABORATORY				
**PARAMETER**	**GRADE 1<br>MILD**	**GRADE 2<br>MODERATE**	**GRADE 3<br>SEVERE**	**GRADE 4<br>POTENTIALLY LIFE-THREATENING**
**HEMATOLOGY**	***Standard International Units are listed in italics***			
Absolute neutrophil count (ANC)				
**Adults**	1,000 – 1,300/mm^3<br>*1.000 x 10^9 – 1.300 x 10^9/L*	750 – 999/mm^3<br>*0.750 x 10^9 – 0.999 x 10^9/L*	500 – 749/mm^3<br>*0.500 x 10^9 – 0.749 x 10^9/L*	< 500/mm^3<br>*< 0.500 x 10^9/L*
Fibrinogen, decreased	100 – 200 mg/dL<br>*1.00 – 2.00 g/L*<br>OR<br>0.75 – 0.99 x LLN	75 – 99 mg/dL<br>*0.75 – 0.99 g/L*<br>OR<br>0.50 – 0.74 x LLN	50 – 74 mg/dL<br>*0.50 – 0.74 g/L*<br>OR<br>0.25 – 0.49 x LLN	< 50 mg/dL<br>*< 0.50 g/L*<br>OR<br>< 0.25 x LLN<br>OR<br>Associated with gross bleeding
Hemoglobin (Hgb)				
**Adults** (HIV <u>POSITIVE</u> ONLY)	8.5 – 10.0 g/dL<br>*1.32 – 1.55 mmol/L*	7.5 – 8.4 g/dL<br>*1.16 – 1.31 mmol/L*	6.50 – 7.4 g/dL<br>*1.01 – 1.15 mmol/L*	< 6.5 g/dL<br>*< 1.01 mmol/L*
International Normalized Ratio of prothrombin time (INR)	1.1 – 1.5 x ULN	1.6 – 2.0 x ULN	2.1 – 3.0 x ULN	> 3.0 x ULN
Methemoglobin	5.0 – 10.0%	10.1 – 15.0%	15.1 – 20.0%	> 20.0%
Prothrombin Time (PT)	1.1 – 1.25 x ULN	1.26 – 1.50 x ULN	1.51 – 3.00 x ULN	> 3.00 x ULN
Partial Thromboplastin Time (PTT)	1.1 – 1.66 x ULN	1.67 – 2.33 x ULN	2.34 – 3.00 x ULN	> 3.00 x ULN
Platelets, decreased	100,000 – 124,999/mm^3<br>*100.000 x 10^9 – 124.999 x 10^9/L*	50,000 – 99,999/mm^3<br>*50.000 x 10^9 – 99.999 x 10^9/L*	25,000 – 49,999/mm^3<br>*25.000 x 10^9 – 49.999 x 10^9/L*	< 25,000/mm^3<br>*< 25.000 x 10^9/L*
WBC, decreased	2,000 – 2,500/mm^3<br>*2.000 x 10^9 – 2.500 x 10^9/L*	1,500 – 1,999/mm^3<br>*1.500 x 10^9 – 1.999 x 10^9/L*	1,000 – 1,499/mm^3<br>*1.000 x 10^9 – 1.499 x 10^9/L*	< 1,000/mm^3<br>*< 1.000 x 10^9/L*
**CHEMISTRIES**	***Standard International Units are listed in italics***			
Acidosis	NA	pH < normal, but ≥ 7.3	pH < 7.3 without life-threatening consequences	pH < 7.3 with life-threatening consequences

LABORATORY				
**PARAMETER**	**GRADE 1 MILD**	**GRADE 2 MODERATE**	**GRADE 3 SEVERE**	**GRADE 4 POTENTIALLY LIFE-THREATENING**
Albumin, serum, low	3.0 g/dL – < LLN<br>*30 g/L – < LLN*	2.0 – 2.9 g/dL<br>*20 – 29 g/L*	< 2.0 g/dL<br>*< 20 g/L*	NA
Alkaline Phosphatase	1.25 – 2.5 x ULN†	2.6 – 5.0 x ULN†	5.1 – 10.0 x ULN†	> 10.0 x ULN†
Alkalosis	NA	pH > normal, but ≤ 7.5	pH > 7.5 without life-threatening consequences	pH > 7.5 with life-threatening consequences
ALT (SGPT)	1.25 – 2.5 x ULN	2.6 – 5.0 x ULN	5.1 – 10.0 x ULN	> 10.0 x ULN
AST (SGOT)	1.25 – 2.5 x ULN	2.6 – 5.0 x ULN	5.1 – 10.0 x ULN	> 10.0 x ULN
Bicarbonate, serum, low	16.0 mEq/L – < LLN<br>*16.0 mmol/L – < LLN*	11.0 – 15.9 mEq/L<br>*11.0 – 15.9 mmol/L*	8.0 – 10.9 mEq/L<br>*8.0 – 10.9 mmol/L*	< 8.0 mEq/L<br>*< 8.0 mmol/L*
Bilirubin (Total)				
**Adults**	1.1 – 1.5 x ULN	1.6 – 2.5 x ULN	2.6 – 5.0 x ULN	> 5.0 x ULN
Calcium, serum, high (corrected for albumin)				
**Adults**	10.6 – 11.5 mg/dL<br>*2.65 – 2.88 mmol/L*	11.6 – 12.5 mg/dL<br>*2.89 – 3.13 mmol/L*	12.6 – 13.5 mg/dL<br>*3.14 – 3.38 mmol/L*	> 13.5 mg/dL<br>*> 3.38 mmol/L*
Calcium, serum, low (corrected for albumin)				
**Adults**	7.8 – 8.4 mg/dL<br>*1.95 – 2.10 mmol/L*	7.0 – 7.7 mg/dL<br>*1.75 – 1.94 mmol/L*	6.1 – 6.9 mg/dL<br>*1.53 – 1.74 mmol/L*	< 6.1 mg/dL<br>*< 1.53 mmol/L*
Cardiac troponin I (cTnI)	NA	NA	NA	Levels consistent with myocardial infarction or unstable angina as defined by the manufacturer
Cardiac troponin T (cTnT)	NA	NA	NA	≥ 0.20 ng/mL<br>OR<br>Levels consistent with myocardial infarction or unstable angina as defined by the manufacturer
Cholesterol (fasting)				
**Adult ≥ 18 years**	200 – 239 mg/dL<br>*5.18 – 6.19 mmol/L*	240 – 300 mg/dL<br>*6.20 – 7.77 mmol/L*	> 300 mg/dL<br>*> 7.77 mmol/L*	NA
Creatine Kinase	3.0 – 5.9 x ULN†	6.0 – 9.9 x ULN†	10.0 – 19.9 x ULN†	≥ 20.0 x ULN†
Creatinine	1.1 – 1.3 x ULN†	1.4 – 1.8 x ULN†	1.9 – 3.4 x ULN†	≥ 3.5 x ULN†
Glucose, serum, high				
Nonfasting	116 – 160 mg/dL<br>*6.44 – 8.88 mmol/L*	161 – 250 mg/dL<br>*8.89 – 13.88 mmol/L*	251 – 500 mg/dL<br>*13.89 – 27.75 mmol/L*	> 500 mg/dL<br>*> 27.75 mmol/L*
Fasting	110 – 125 mg/dL<br>*6.11 – 6.94 mmol/L*	126 – 250 mg/dL<br>*6.95 – 13.88 mmol/L*	251 – 500 mg/dL<br>*13.89 – 27.75 mmol/L*	> 500 mg/dL<br>*> 27.75 mmol/L*
Glucose, serum, low				
**Adults**	55 – 64 mg/dL<br>*3.05 – 3.55 mmol/L*	40 – 54 mg/dL<br>*2.22 – 3.06 mmol/L*	30 – 39 mg/dL<br>*1.67 – 2.23 mmol/L*	< 30 mg/dL<br>*< 1.67 mmol/L*

LABORATORY				
**PARAMETER**	**GRADE 1 MILD**	**GRADE 2 MODERATE**	**GRADE 3 SEVERE**	**GRADE 4 POTENTIALLY LIFE-THREATENING**
Lactate	< 2.0 x ULN without acidosis	≥ 2.0 x ULN without acidosis	Increased lactate with pH < 7.3 without life-threatening consequences	Increased lactate with pH < 7.3 with life-threatening consequences
LDL cholesterol (fasting)				
**Adult ≥ 18 years**	130 – 159 mg/dL<br>*3.37 – 4.12 mmol/L*	160 – 190 mg/dL<br>*4.13 – 4.90 mmol/L*	≥ 190 mg/dL<br>*≥ 4.91 mmol/L*	NA
Lipase	1.1 – 1.5 x ULN	1.6 – 3.0 x ULN	3.1 – 5.0 x ULN	> 5.0 x ULN
Magnesium, serum, low	1.2 – 1.4 mEq/L<br>*0.60 – 0.70 mmol/L*	0.9 – 1.1 mEq/L<br>*0.45 – 0.59 mmol/L*	0.6 – 0.8 mEq/L<br>*0.30 – 0.44 mmol/L*	< 0.60 mEq/L<br>*< 0.30 mmol/L*
Pancreatic amylase	1.1 – 1.5 x ULN	1.6 – 2.0 x ULN	2.1 – 5.0 x ULN	> 5.0 x ULN
Phosphate, serum, low				
**Adults**	2.5 mg/dL – < LLN<br>*0.81 mmol/L – < LLN*	2.0 – 2.4 mg/dL<br>*0.65 – 0.80 mmol/L*	1.0 – 1.9 mg/dL<br>*0.32 – 0.64 mmol/L*	< 1.00 mg/dL<br>*< 0.32 mmol/L*
Potassium, serum, high	5.6 – 6.0 mEq/L<br>*5.6 – 6.0 mmol/L*	6.1 – 6.5 mEq/L<br>*6.1 – 6.5 mmol/L*	6.6 – 7.0 mEq/L<br>*6.6 – 7.0 mmol/L*	> 7.0 mEq/L<br>*> 7.0 mmol/L*
Potassium, serum, low	3.0 – 3.4 mEq/L<br>*3.0 – 3.4 mmol/L*	2.5 – 2.9 mEq/L<br>*2.5 – 2.9 mmol/L*	2.0 – 2.4 mEq/L<br>*2.0 – 2.4 mmol/L*	< 2.0 mEq/L<br>*< 2.0 mmol/L*
Sodium, serum, high	146 – 150 mEq/L<br>*146 – 150 mmol/L*	151 – 154 mEq/L<br>*151 – 154 mmol/L*	155 – 159 mEq/L<br>*155 – 159 mmol/L*	≥ 160 mEq/L<br>*≥ 160 mmol/L*
Sodium, serum, low	130 – 135 mEq/L<br>*130 – 135 mmol/L*	125 – 129 mEq/L<br>*125 – 129 mmol/L*	121 – 124 mEq/L<br>*121 – 124 mmol/L*	≤ 120 mEq/L<br>*≤ 120 mmol/L*
Triglycerides (fasting)	NA	500 – 750 mg/dL<br>*5.65 – 8.48 mmol/L*	751 – 1,200 mg/dL<br>*8.49 – 13.56 mmol/L*	> 1,200 mg/dL<br>*> 13.56 mmol/L*
Uric acid	7.5 – 10.0 mg/dL<br>*0.45 – 0.59 mmol/L*	10.1 – 12.0 mg/dL<br>*0.60 – 0.71 mmol/L*	12.1 – 15.0 mg/dL<br>*0.72 – 0.89 mmol/L*	> 15.0 mg/dL<br>*> 0.89 mmol/L*
**URINALYSIS**	***Standard International Units are listed in italics***			
Hematuria (microscopic)	6 – 10 RBC/HPF	> 10 RBC/HPF	Gross, with or without clots OR with RBC casts	Transfusion indicated
Proteinuria, random collection	1 +	2 – 3 +	4 +	NA
Proteinuria, 24 hour collection				
**Adults**	200 – 999 mg/24 h<br>*0.200 – 0.999 g/d*	1,000 – 1,999 mg/24 h<br>*1.000 – 1.999 g/d*	2,000 – 3,500 mg/24 h<br>*2.000 – 3.500 g/d*	> 3,500 mg/24 h<br>*> 3.500 g/d*

ANEXO 5

DECLARACION DE HELSINKI DE LA ASOCIACION MEDICA MUNDIAL

Principios éticos para las investigaciones médicas en seres humanos

- Adoptada por la 18ª Asamblea Médica Mundial - Helsinki, Finlandia, junio 1964 y enmendada por la 29ª Asamblea Médica Mundial - Tokio, Japón, octubre 1975
- 35ª Asamblea Médica Mundial - Venecia, Italia, octubre 1983
- 41ª Asamblea Médica Mundial - Hong Kong, septiembre 1989
- 48ª Asamblea General - Somerset West, Suráfrica, octubre 1996 y la
- 52ª Asamblea General - Edimburgo, Escocia, octubre 2000

A. INTRODUCCION

1. La Asociación Médica Mundial ha promulgado la Declaración de Helsinki como una propuesta de principios éticos que sirvan para orientar a los médicos y a otras personas que realizan investigación médica en seres humanos. La investigación médica en seres humanos incluye la investigación del material humano o de información identificables.

2. El deber del médico es promover y velar por la salud de las personas. Los conocimientos y la conciencia del médico han de subordinarse al cumplimiento de ese deber.

3. La Declaración de Ginebra de la Asociación Médica Mundial vincula al médico con la fórmula "velar solícitamente y ante todo por la salud de mi paciente", y el Código Internacional de Ética Médica afirma que: "El médico debe actuar solamente en el interés del paciente al proporcionar atención médica que pueda tener el efecto de debilitar la condición mental y física del paciente".

4. El progreso de la medicina se basa en la investigación, la cual, en último término, tiene que recurrir muchas veces a la experimentación en seres humanos.

5. En investigación médica en seres humanos, la preocupación por el bienestar de los seres humanos debe tener siempre primacía sobre los intereses de la ciencia y de la sociedad.

6. El propósito principal de la investigación médica en seres humanos es mejorar los procedimientos preventivos, diagnósticos y terapéuticos, y también comprender la etiología y patogenia de las enfermedades. Incluso, los mejores métodos preventivos, diagnósticos y terapéuticos disponibles deben ponerse a prueba continuamente a través de la investigación para que sean eficaces, efectivos, accesibles y de calidad.

7. En la práctica de la medicina y de la investigación médica del presente, la mayoría de los procedimientos preventivos, diagnósticos y terapéuticos implican algunos riesgos y costos.

8. La investigación médica está sujeta a normas éticas que sirven para promover el respeto a todos los seres humanos y para proteger su salud y sus derechos individuales. Algunas poblaciones sometidas a la investigación son vulnerables y necesitan protección especial. Se deben reconocer las necesidades particulares de los que tienen desventajas económicas y médicas. También se debe prestar atención especial a los que no pueden otorgar o rechazar el consentimiento por sí mismos, a los que pueden otorgar el consentimiento bajo presión, a los que no se beneficiarán personalmente con la investigación y a los que tienen la investigación combinada con la atención médica.

9. Los investigadores deben conocer los requisitos éticos, legales y jurídicos para la investigación en seres humanos en sus propios países, al igual que los requisitos internacionales vigentes. No se debe permitir que

un requisito ético, legal o jurídico disminuya o elimine cualquiera medida de protección para los seres humanos establecida en esta Declaración.

B. PRINCIPIOS BASICOS PARA TODA INVESTIGACION MEDICA

10. En la investigación médica, es deber del médico proteger la vida, la salud, la intimidad y la dignidad del ser humano.

11. La investigación médica en seres humanos debe conformarse con los principios científicos generalmente aceptados, y debe apoyarse en un profundo conocimiento de la bibliografía científica, en otras fuentes de información pertinentes, así como en experimentos de laboratorio correctamente realizados y en animales, cuando sea oportuno.

12. Al investigar, hay que prestar atención adecuada a los factores que puedan perjudicar el medio ambiente. Se debe cuidar también del bienestar de los animales utilizados en los experimentos.

13. El proyecto y el método de todo procedimiento experimental en seres humanos debe formularse claramente en un protocolo experimental. Este debe enviarse, para consideración, comentario, consejo, y cuando sea oportuno, aprobación, a un comité de evaluación ética especialmente designado, que debe ser independiente del investigador, del patrocinador o de cualquier otro tipo de influencia indebida. Se sobrentiende que ese comité independiente debe actuar en conformidad con las leyes y reglamentos vigentes en el país donde se realiza la investigación experimental. El comité tiene el derecho de controlar los ensayos en curso. El investigador tiene la obligación de proporcionar información del control al comité, en especial sobre todo incidente adverso grave. El investigador también debe presentar al comité, para que la revise, la información sobre financiamiento, patrocinadores, afiliaciones institucionales, otros posibles conflictos de interés e incentivos para las personas del estudio.

14. El protocolo de la investigación debe hacer referencia siempre a las consideraciones éticas que fueran del caso, y debe indicar que se han observado los principios enunciados en esta Declaración.

15. La investigación médica en seres humanos debe ser llevada a cabo sólo por personas científicamente calificadas y bajo la supervisión de un médico clínicamente competente. La responsabilidad de los seres humanos debe recaer siempre en una persona con capacitación médica, y nunca en los participantes en la investigación, aunque hayan otorgado su consentimiento.

16. Todo proyecto de investigación médica en seres humanos debe ser precedido de una cuidadosa comparación de los riesgos calculados con los beneficios previsibles para el individuo o para otros. Esto no impide la participación de voluntarios sanos en la investigación médica. El diseño de todos los estudios debe estar disponible para el público.

17. Los médicos deben abstenerse de participar en proyectos de investigación en seres humanos a menos que estén seguros de que los riesgos inherentes han sido adecuadamente evaluados y de que es posible hacerles frente de manera satisfactoria. Deben suspender el experimento en marcha si observan que los riesgos que implican son más importantes que los beneficios esperados o si existen pruebas concluyentes de resultados positivos o benéficos.

18. La investigación médica en seres humanos sólo debe realizarse cuando la importancia de su objetivo es mayor que el riesgo inherente y los costos para el individuo. Esto es especialmente importante cuando los seres humanos son voluntarios sanos.

19. La investigación médica sólo se justifica si existen posibilidades razonables de que la población, sobre la que la investigación se realiza, podrá beneficiarse de sus resultados.

20. Para tomar parte en un proyecto de investigación, los individuos deben ser participantes voluntarios e informados.

21. Siempre debe respetarse el derecho de los participantes en la investigación a proteger su integridad. Deben tomarse toda clase de precauciones para resguardar la intimidad de los individuos, la confidencialidad de la información del paciente y para reducir al mínimo las consecuencias de la investigación sobre su integridad física y mental y su personalidad.

22. En toda investigación en seres humanos, cada individuo potencial debe recibir información adecuada acerca de los objetivos, métodos, fuentes de financiamiento, posibles conflictos de intereses, afiliaciones institucionales del investigador, beneficios calculados, riesgos previsibles e incomodidades derivadas del experimento. La persona debe ser informada del derecho de participar o no en la investigación y de retirar su consentimiento en cualquier momento, sin exponerse a represalias. Después de asegurarse de que el individuo ha comprendido la información, el médico debe obtener entonces, preferiblemente por escrito, el consentimiento informado y voluntario de la persona. Si el consentimiento no se puede obtener por escrito, el proceso para obtenerlo debe ser documentado formalmente ante testigos.

23. Al obtener el consentimiento informado para el proyecto de investigación, el médico debe poner especial cuidado cuando el individuo está vinculado con él por una relación de dependencia o si consiente bajo presión. En un caso así, el consentimiento informado debe ser obtenido por un médico bien informado que no participe en la investigación y que nada tenga que ver con aquella relación.

24. Cuando la persona sea legalmente incapaz, o inhábil física o mentalmente de otorgar consentimiento, o menor de edad, el investigador debe obtener el consentimiento informado del representante legal y de acuerdo con la ley vigente. Estos grupos no deben ser incluidos en la investigación a menos que ésta sea necesaria para promover la salud de la población representada y esta investigación no pueda realizarse en personas legalmente capaces.

25. Si una persona considerada incompetente por la ley, como es el caso de un menor de edad, es capaz de dar su asentimiento a participar o no en la investigación, el investigador debe obtenerlo, además del consentimiento del representante legal.

26. La investigación en individuos de los que no se puede obtener consentimiento, incluso por representante o con anterioridad, se debe realizar sólo si la condición física / mental que impide obtener el consentimiento informado es una característica necesaria de la población investigada. Las razones específicas por las que se utilizan participantes en la investigación que no pueden otorgar su consentimiento informado deben ser estipuladas en el protocolo experimental que se presenta para consideración y aprobación del comité de evaluación. El protocolo debe establecer que el consentimiento para mantenerse en la investigación debe obtenerse a la brevedad posible del individuo o de un representante legal.

27. Tanto los autores como los editores tienen obligaciones éticas. Al publicar los resultados de su investigación, el médico está obligado a mantener la exactitud de los datos y resultados. Se deben publicar tanto los resultados negativos como los positivos o de lo contrario deben estar a la disposición del público. En la publicación se debe citar la fuente de financiamiento, afiliaciones institucionales y cualquier posible conflicto de intereses. Los informes sobre investigaciones que no se ciñan a los principios descritos en esta Declaración no deben ser aceptados para su publicación.

C. PRINCIPIOS APLICABLES CUANDO LA INVESTIGACION MEDICA SE COMBINAN CON LA ATENCION MEDICA

28. El médico puede combinar la investigación médica con la atención médica, sólo en la medida en que tal investigación acredite un justificado valor potencial preventivo, diagnóstico o terapéutico. Cuando la investigación médica se combina con la atención médica, las normas adicionales se aplican para proteger a los pacientes que participan en la investigación.
29. Los posibles beneficios, riesgos, costos y eficacia de todo procedimiento nuevo deben ser evaluados mediante su comparación con los mejores métodos preventivos, diagnósticos y terapéuticos disponibles. Ello no excluye que pueda usarse un placebo, o ningún tratamiento, en estudios para los que no se dispone de procedimientos preventivos, diagnósticos o terapéuticos probados.
30. Al final de la investigación, todos los pacientes que participan en el estudio deben tener la certeza de que contarán con los mejores métodos preventivos, diagnósticos y terapéuticos disponibles, identificados por el estudio.
31. El médico debe informar cabalmente al paciente los aspectos de la atención que tienen relación con la investigación. La negativa del paciente a participar en una investigación nunca debe perturbar la relación médico-paciente.
32. Cuando los métodos preventivos, diagnósticos o terapéuticos disponibles han resultado ineficaces en la atención de un enfermo, el médico, con el consentimiento informado del paciente, puede permitirse usar procedimientos preventivos, diagnósticos y terapéuticos nuevos o no probados, si, a su juicio, ello da alguna esperanza de salvar la vida, restituir la salud o aliviar el sufrimiento. Siempre que sea posible, tales medidas deben ser investigadas a fin de evaluar su seguridad y eficacia. En todos los casos, esa información nueva debe ser registrada y, cuando sea oportuno, publicada. Se deben seguir todas las otras normas pertinentes de esta Declaración.

ANEXO 6

Principios de Buena práctica clínica y garantía de calidad

De acuerdo con la legislación del Estado (R.D. 561/1993)[10] y de la Directiva Comunitaria (91/507/CEE)[11], todos los ensayos clínicos en todas sus fases, incluyendo aquellos de biodisponibilidad y bioequivalencia, se realizarán según las normas de buena práctica clínica.

Las normas de buena práctica clínica (BPC) son una ayuda para asegurar que la investigación clínica se lleva a cabo según los más elevados estándares de calidad, siguiendo los criterios éticos (basados en la Declaración de Helsinki y desarrollos ulteriores) y con un equipo médico de garantía. En este sentido, las normas de BPC constituyen una norma para el diseño, dirección, realización, cumplimiento, monitorización, auditoría, registro, análisis e información de ensayos clínicos que asegura que los datos y resultados obtenidos son correctos y creíbles y que se protegen los derechos, integridad y confidencialidad de los sujetos del ensayo.

Los principios de la BPC (buena práctica clínica) son los siguientes:

1. Los ensayos clínicos deben ser realizados de acuerdo con los principios éticos que tienen su origen en la Declaración Helsinki, los cuales son consistentes con la BPC y los requisitos reguladores pertinentes.

2. Antes de iniciar un ensayo, deben considerarse las inconveniencias y riesgos previsibles en relación con el beneficio previsto paras el sujeto individual del ensayo y para la sociedad. Un ensayo debe ser iniciado y continuado sólo si los beneficios previstos justifican los riesgos.

3. Los derechos, seguridad y bienestar de los sujetos del ensayo son las consideraciones más importantes y deben prevalecer sobre los intereses de la ciencia y la sociedad.

4. La información clínica y no clínica disponible sobre un producto en investigación debe ser adecuada para respaldar el ensayo clínico propuesto.

5. Los ensayos clínicos deben ser científicamente razonables y estar descritos en un protocolo claro y detallado.

6. Un ensayo debe ser realizado de acuerdo con el protocolo, que ha recibido una revisión previa y una opinión favorable/aprobación de un Consejo Institucional de Revisión (CIR) / Comité Ético Independiente (CEI).

7. El cuidado médico que reciben los sujetos y las decisiones médicas que afectan a los mismos deben ser siempre responsabilidad de un médico cualificado o, en su caso de un odontólogo cualificado.

8. Cada individuo implicado en la realización de un ensayo debe ser cualificado, por educación, formación y experiencia, para realizar su labor.

9. Se debe obtener un consentimiento informado, dado libremente, de cada sujeto, previamente a su participación en el ensayo clínico.

10. Toda la información del ensayo clínico debe ser registrada, manejada y almacenada de forma que permita su comunicación, verificación e interpretación exactas.

11. Debe protegerse la confidencialidad de los registros que pudieran identificar a los sujetos, respetando la privacidad y las normas de confidencialidad de acuerdo con los requisitos reguladores pertinentes.

12. Los productos en investigación deben ser fabricados, manejos y almacenados de acuerdo con las normas de buena práctica de fabricación pertinentes y se deben utilizar de acuerdo con el protocolo aprobado.

Se deberán llevar a cabo los sistemas y procedimientos que aseguren la calidad de cada aspecto del ensayo.

ANEXO 7

POLIZA DE SEGURO

HDI

SEGUROS

CERTIFICADO

La Compañía **HDI HANNOVER International (España), Seguros y Reaseguros, S.A.**, con domicilio en **Madrid, c/ Luchana, 23, 6ª**,

CERTIFICA

Que tiene contratada y en vigor la póliza de Responsabilidad Civil **Nº 130/001/004827** referida a ensayos clínicos, siendo el Tomador del Seguro el **DR. LUIS FERNANDO LOPEZ CORTES**.

La póliza mencionada cubre la Responsabilidad Civil que pueda derivarse por la realización del siguiente ensayo clínico:

Titulo: **"Eficacia de dosis bajas de interferón pegilado alfa-2a junto con ribavirina en el tratamiento de la hepatopatía crónica por virus C (genotipos 2 o 3) en pacientes coinfectados por el VIH."**

Código de Protocolo: SAEI_IFN_1

Ctro. Inv.: **H. U. Virgen del Rocio de Sevilla.**

Inv. Principal: **Dr. Luis F. López Cortés.**

El ensayo se experimenta en territorio nacional conforme a lo establecido en el Real Decreto 223/2004 de 6 de Febrero, publicado en el B.O.E. con fecha 7 de febrero del mismo año, que la desarrolla, estableciéndose un limite de la suma asegurada de € 3.000.000 por año así como un sublímite de € 250.000 por sujeto.

Que se encuentra cubierta la totalidad de la duración del ensayo, previsto para el periodo 01.09.2007 – 31.12.2009.

Y para que así conste, se expide el presente Certificado, en Madrid, a 16 de febrero de 2007.

HDI HANNOVER INTERNATIONAL ESPAÑA
Cía. de Seguros y Reaseguros, S.A.

HDI HANNOVER INTERNATIONAL (ESPAÑA)
SEGUROS Y REASEGUROS, S.A.
Luchana, 23
28010 MADRID

Tel. 91 444 20 00
Fax 91 444 20 19
E-mail: hie@hdi.es

Banco Popular Español
Cta. Cte. 0075 1083 71 060 00412 06
IBAN ES 45 0075 1083 71 060 00412 06
SWIFT CODE: POPUESMM

HDI

SEGUROS

CERTIFICADO

La Compañía **HDI HANNOVER International (España), Seguros y Reaseguros, S.A.**, con domicilio en **Madrid, c/ Luchana, 23, 6ª**,

CERTIFICA

Que tiene contratada y en vigor la póliza de Responsabilidad Civil **Nº 130/001/004827** referida a ensayos clínicos, siendo el Tomador del Seguro el **DR. LUIS FERNANDO LOPEZ CORTES**.

La póliza mencionada cubre la Responsabilidad Civil que pueda derivarse por la realización del siguiente ensayo clínico:

Título: **"Eficacia de dosis bajas de interferón pegilado alfa-2a junto con ribavirina en el tratamiento de la hepatopatía crónica por virus C (genotipos 2 o 3) en pacientes coinfectados por el VIH."**

Código de Protocolo: SAEI_IFN_1

Ctro. Inv.: **H.U. de Valme de Sevilla.**

Inv. Principal: **Dr. Angel Garcia Garcia.**

El ensayo se experimenta en territorio nacional conforme a lo establecido en el Real Decreto 223/2004 de 6 de Febrero, publicado en el B.O.E. con fecha 7 de febrero del mismo año, que la desarrolla, estableciéndose un límite de la suma asegurada de € 3.000.000 por año así como un sublímite de € 250.000 por sujeto.

Que se encuentra cubierta la totalidad de la duración del ensayo, previsto para el periodo 01.09.2007 – 31.12.2009.

Y para que así conste, se expide el presente Certificado, en Madrid, a 16 de febrero de 2007.

HDI HANNOVER INTERNATIONAL ESPAÑA
[illegible] Seguros y Reaseguros, S.A.

HDI HANNOVER INTERNATIONAL (ESPAÑA)
SEGUROS Y REASEGUROS, S.A.
Luchana, 23
28010 MADRID

Tel. 91 444 20 00
Fax 91 444 20 19
E-mail: hie@hdi.es

Banco Popular Español
Cta. Cte. 0075 1083 71 060 00412 06
IBAN ES 45 0075 1083 71 060 00412 06
SWIFT CODE: POPUESMM

HDI

SEGUROS

CERTIFICADO

La Compañía **HDI HANNOVER International (España), Seguros y Reaseguros, S.A.**, con domicilio en **Madrid, c/ Luchana, 23, 6ª**,

CERTIFICA

Que tiene contratada y en vigor la póliza de Responsabilidad Civil **Nº 130/001/004827** referida a ensayos clínicos, siendo el Tomador del Seguro el **DR. LUIS FERNANDO LOPEZ CORTES**.

La póliza mencionada cubre la Responsabilidad Civil que pueda derivarse por la realización del siguiente ensayo clínico:

Título: **"Eficacia de dosis bajas de interferón pegilado alfa-2a junto con ribavirina en el tratamiento de la hepatopatia crónica por virus C (genotipos 2 o 3) en pacientes coinfectados por el VIH."**

Código de Protocolo: SAEI_IFN_1

Ctro. Inv.: **H.U. Reina Sofia de Córdoba.**

Inv. Principal: **Dr. Antonio Rivero Román.**

El ensayo se experimenta en territorio nacional conforme a lo establecido en el Real Decreto 223/2004 de 6 de Febrero, publicado en el B.O.E. con fecha 7 de febrero del mismo año, que la desarrolla, estableciéndose un límite de la suma asegurada de € 3.000.000 por año así como un sublímite de € 250.000 por sujeto.

Que se encuentra cubierta la totalidad de la duración del ensayo, previsto para el periodo 01.09.2007 – 31.12.2009.

Y para que así conste, se expide el presente Certificado, en Madrid, a 16 de febrero de 2007.

HDI HANNOVER INTERNATIONAL ESPAÑA
Cía de Seguros y Reaseguros, S.A.

Autorizada por O.M. de fecha 22-12-1959, inscrita en el Registro de Entidades Aseguradoras Nº C - 726. C.I.F. A81250243.

HDI HANNOVER INTERNATIONAL (ESPAÑA)
SEGUROS Y REASEGUROS, S.A.
Luchana, 23
28010 MADRID

Tel. 91 444 20 00
Fax 91 444 20 19
E-mail: hie@hdi.es

Banco Popular Español
Cta. Cte. 0075 1083 71 060 00412 06
IBAN ES 45 0075 1083 71 060 00412 06
SWIFT CODE: POPUESMM

ANEXO 8

CUADERNO DE RECOGIDA DE DATOS

Eficacia de dosis bajas de interferón pegilado alfa-2a junto con ribavirina en el tratamiento de la hepatopatía crónica por virus C (genotipos 2 o 3) en pacientes coinfectados por el VIH

Código del protocolo: SAE_IFN_1

Nº EudraCT: 2007-000814-35

Versión 11-02-2007

INICIALES PACIENTE _ _ _ _<br>CODIGO Nº _ _. _ _<br>CENTRO ______________________________

**Fecha de nacimiento: / /**	**Edad:**

Fecha de inclusión y firma del investigador

INICIALES: **CODIGO:** **CENTRO:**

Datos infección VIH a la inclusión

Estadío previo (CDC):

Factor riesgo:

CD4/µl nadir: **fecha:**

CD4/µl: **fecha:**

RNA-VIH/ml: **fecha:**

Tratamiento antirretroviral actual:

fecha de inicio:

Datos hepatitis crónica y/o cirrosis por virus C en la inclusión

Genotipo:

Carga viral C: UI/ml

Biopsia hepática (fecha y resultado en caso de haberse practicado)**:**

Peso: kg

Tratamiento prescrito:

Comentarios a la inclusión o durante el seguimiento:

	Fecha dd/mm/aa	Dosis Peg-IFN-α	Dosis Rbv	Peso Kg	RNA-VHC UI/ml	CD4 /µl	RNA-VIH copias/ml	Hb g/dl	PMN /µl	Linfocitos /µl	Plaquetas /µl	Creatinina mg/dl	Cl creatinina mL/min	GPT UI/ml	TSH	Cp IFN	Cp Rbv
Inclusión	X	X	X	X	X	X	X	X	X	X	X	X	X	X	X		
1ª semana	X	X	X	X	X			X	X	X	X	X	X	X	X	X	X
4ª semana	X	X	X	X	X	X	X	X	X	X	X	X	X	X	X	X	X
12ª semana	X	X	X	X	X	X	X	X	X	X	X	X	X	X	X	X	X
24ª semana	X	X	X	X	X	X	X	X	X	X	X	X	X	X	X	X	X
36ª semana	X	X	X	X		X	X	X	X	X	X	X	X	X		X	X

EFECTOS ADVERSOS: SI/ NO. En caso de respuesta afirmativa, fecha y describir según grado:

GRADO: 1- Leve/s

2- Moderado/s

3- Grave/s

4- Obligan a suspender tratamiento. **SI/ NO.** **Fecha de suspensión:**

Modificaciones de dosis de fármacos (describir y fecha):

ANEXO 9

MEMORIA ECONÓMICA

Personal .... 84.000,00 €

(Equivalente a contrato para 1 licenciado durante toda la duración del ensayo (28.000,00 €/año))

Seguro de Responsabilidad Civil .... 7.500,00 €

Determinaciones de Interferón (x 350) .... 6.507,00 €

(9 kits, 40 determinaciones/kit, de ELISA Hu-INF-α® -PBL Biomedical Laboratories, New Jersey. USA x 723,00 €)

Determinaciones de Ribavirina (15, 00 € x 350) .... 5.250,00 €

Desplazamientos de monitorización .... 6.000,00 €

Desplazamientos y reuniones investigadores .... 3.000,00 €

Transporte de muestras .... 1.500,00 €

TOTAL: ....113.757,00 €

ANEXO 10

FICHA TECNICA DEL PRODUCTO

Se adjunta en fichero *pdf
